# A new antiviral scaffold for human norovirus identified with computer-aided approaches on the viral polymerase

**DOI:** 10.1038/s41598-019-54903-7

**Published:** 2019-12-05

**Authors:** Gilda Giancotti, Ilaria Rigo, Gaia Pasqualetto, Mark T. Young, Johan Neyts, Joana Rocha-Pereira, Andrea Brancale, Salvatore Ferla, Marcella Bassetto

**Affiliations:** 1 Cardiff School of Pharmacy and Pharmaceutical Sciences, Cardiff, King Edward VII Avenue, Cardiff, CF103NB UK; 2 Cardiff School of Biosciences, Sir Martin Evans Building, Museum Avenue, Cardiff, CF10 3AX UK; 3 KU Leuven – Department of Microbiology, Immunology and Transplantation, Rega Institute, Laboratory of Virology and Chemotherapy, Leuven, Belgium; 4 Department of Chemistry, Swansea University, Swansea, UK

**Keywords:** Drug discovery and development, Structure-based drug design

## Abstract

Human norovirus is the leading cause of acute gastroenteritis worldwide, affecting every year 685 million people. In about one third of cases, this virus affects children under five years of age, causing each year up to 200,000 child deaths, mainly in the developing countries. Norovirus outbreaks are associated with very significant economic losses, with an estimated societal cost of 60 billion dollars per year. Despite the marked socio-economic consequences associated, no therapeutic options or vaccines are currently available to treat or prevent this infection. One promising target to identify new antiviral agents for norovirus is the viral polymerase, which has a pivotal role for the viral replication and lacks closely homologous structures in the host. Starting from the scaffold of a novel class of norovirus polymerase inhibitors recently discovered in our research group with a computer-aided method, different new chemical modifications were designed and carried out, with the aim to identify improved agents effective against norovirus replication in cell-based assays. While different new inhibitors of the viral polymerase were found, a further computer-aided ligand optimisation approach led to the identification of a new antiviral scaffold for norovirus, which inhibits human norovirus replication at low-micromolar concentrations.

## Introduction

Human norovirus (HuNoV) is the main cause of acute gastroenteritis worldwide, leading to approximately 200,000 deaths every year, with an associated societal cost of 60 billion USD^1^. While infection with this virus is responsible for a self-limiting disease in healthy individuals, the condition often becomes severe or life-threatening in immune-compromised patients, children and the elderly^2,3^. Despite the serious health and economic consequences associated, no therapeutic options or vaccines are currently available^4^, attracting significant research efforts into the identification of antiviral candidates, to be used to treat the infection and as a prophylactic measure in the case of outbreaks. Research into much needed anti-norovirus agents has been hampered by several factors, including the inaccessibility of a cell-culture human norovirus propagation system until recently. Despite this, there are ways to study the antiviral effect of molecules by using murine norovirus (MNV), which represents a good surrogate, particularly regarding the viral enzymes involved in replication, and a HuNoV replicon cell line^5,6^. Currently, only a few antiviral agents are under evaluation in pre-clinical and clinical stages. In particular, two small-molecules are currently under clinical investigations: Nitazoxanide, an antiprotozoal agent which reduces the duration and alleviates the symptoms of norovirus infection with a still unknown mechanism of action^7,8^, and CMX521, a nucleoside analogue which shows efficacy against multiple viral genotypes^9^. Other therapeutic candidates in pre-clinical development include different agents targeting mainly the viral polymerase or protease, due to their essential roles in the viral replication and the absence of homologous host enzymes^10–17^.

**Figure 1:**
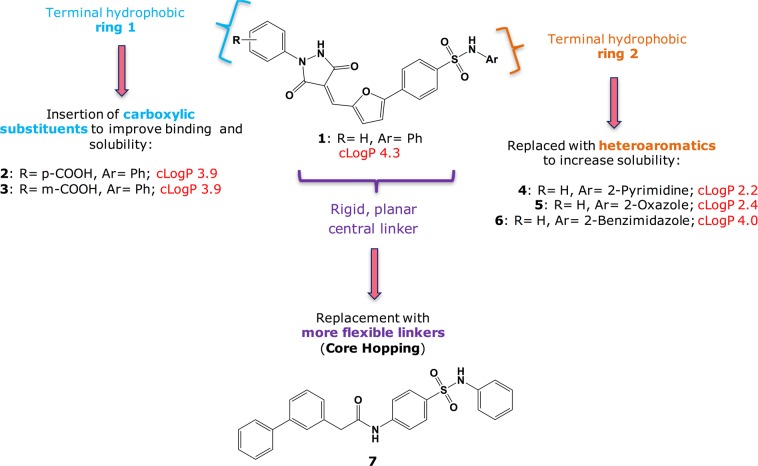
Structural features of previous hit **1** and strategies for the rational/computer-aided modification of its scaffold.

As a member of the *Caliciviridae* family, norovirus is characterised by a single-stranded positive-sense RNA genome, which is replicated by the viral RNA-dependent RNA-polymerase (RdRp) function located in the viral non-structural protein NS7^18^. As revealed by crystallographic data, norovirus polymerase structure highly resembles the one of other positive-strand RNA viruses^19^, and its activity of RNA synthesis can be initiated *de novo* RNA or via a VPg-primed mechanism^20^. Due to its essential role in the viral replication, and to the repeatedly proven success of targeting viral polymerases in antiviral drug discovery^21^, norovirus RdRp has been previously chosen in our research group as a promising target for the identification of new anti-norovirus agents, focussing in particular on the identification of novel non-nucleoside inhibitors (NNIs) of this enzyme. A limited number of inhibitors of this type has been reported so far for norovirus RdRp, but the majority of these compounds lack any activity against the viral replication in cellular systems, possibly due to poor cell permeability and drug-like properties^22^. As several crystal structures are available for human and murine norovirus polymerase, including ternary complexes with nucleotide analogues and with allosteric non-nucleoside inhibitors^23–28^, the study of these structures has been the starting point for a structure-based virtual screening study that led to the identification of our broad-spectrum *Caliciviridae* RdRp inhibitor **1** (Fig. 1)^29^. As is the case for other reported NNIs of norovirus RdRp, despite showing an enzyme inhibition in the low micromolar range, **1** was associated with a very mild effect against norovirus replication in cell-based systems, possibly due to its poor aqueous solubility. Moreover, this compound showed some cytotoxicity at relatively low concentrations, with a CC_50_ of ~64 μM, possibly due, at least in part, to its low solubility and precipitation from the assay medium.

In the present study, the structure of **1** has been rationally modified in order to improve its drug-like properties and achieve an antiviral effect against norovirus replication in cell-systems. The novel structural modifications carried out have allowed a better understanding of the functional groups required for enzymatic and antiviral activity, and the successful identification of a new anti-norovirus scaffold with antiviral EC_50_ values in the low micromolar range. This new scaffold represents a promising starting point for further optimisations and for the potential development of a viable treatment for norovirus infections.

## Results and Discussion

### Rational modifications on compound 1

**1** is characterised by a central 5-phenylfuran-2-ylmethylene-pyrazolidine-3,5-dione core (planar central linker in Fig. 1), substituted at position 1 of the pyrazolidine with a benzene ring (terminal hydrophobic ring 1), and at position 4 of the phenyl ring with a N-phenylsulfonamide (terminal hydrophobic ring 2). These structural features render **1** relatively hydrophobic (calculated logP (o/w) 4.3) and poorly soluble, limiting its potential as a drug.

As described by Hashimoto *et al*.^30^, decreasing the logP of a molecule by introducing a hydrophilic group is a classical and general strategy for improving its aqueous solubility. Following this rationale, two main structural modifications have been planned for **1** to achieve this result: 1) the introduction of a carboxylic acid group at different positions of terminal hydrophobic ring 1 (compounds **2** and **3** in Fig. 1), which should also provide the potential for extra interactions with the RdRp active site; 2) the replacement of the terminal hydrophobic ring 2 with different heteroaromatics (compounds **4–6** in Fig. 1). In particular, this second approach was designed as a consequence of our previously published work^29^, in which a thiazole derivative of **1** showed a better antiviral activity in a cell-based assay (40% inhibition of murine norovirus (MNV)-induced plaque formation at 10 μM), without showing significant solubility issues, but was found to be relatively cytotoxic, with a low selectivity index. Both types of modifications increase the polarity of the original molecule, which could influence in a positive manner their antiviral activity against norovirus replication in cell-based systems. Moreover, as suggested above, the new modifications may improve the binding to the viral polymerase, better mimicking the polar phosphate groups present in the RNA natural substrate of this enzyme.

**Figure 2:**
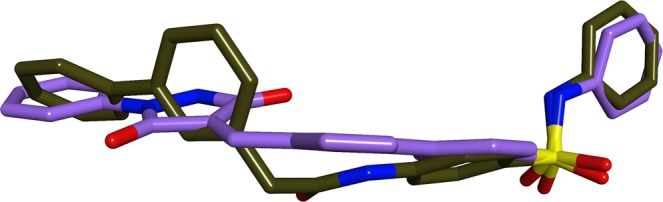
Three-dimensional superimposition, calculated with the Alignment tool in MOE 2018^37^, of the energetically minimised structures of **1**, with carbon atoms in lilac, and **7**, with carbon atoms in green-uniform. The new linker inserted in **7** significantly reduces the rigidity and planarity of the linker in **1**.

Another feature that is recognised to influence compound solubility in a negative fashion is molecular planarity, which enhances crystal packing: poorly soluble compounds are often found to form very stable crystals, which are difficult to be broken by the solvent molecules^31^. Disruption of molecular planarity is usually reflected in improved solubility. As the structure of **1** is predominantly planar, an alternative strategy to reduce its solubility issues was to attempt to disrupt its planarity, by applying a scaffold replacement procedure using Schrödinger Core Hopping tool^32^. During this analysis, the two terminal hydrophobic rings were kept as fixed, while searching for alternative groups to replace the central planar core in **1**. Among the different modifications generated by the program, a *N*-2-diphenylacetamide group was chosen as a more flexible linker to replace the phenylfuran-2-ylmethylene-pyrazolidine-3,5-dione core, and the new compound **7** (Fig. 1) was designed. The new linker selection was driven by an overall molecular length preservation and synthetic accessibility. Despite penalising the new compound from a logP point of view (calculated logP (o/w) 5.3), the effect of the new central core on disrupting the planarity of **1** appears to be significant, as shown in Fig. 2.

## Chemistry

### Synthesis of compounds with modifications on the two terminal hydrophobic rings

A seven-step synthetic pathway previously developed and optimised by our research group has been used for the preparation of a small family of seven new derivatives bearing modifications on the terminal hydrophobic ring 1 or on ring 2^29^.

**Figure 3:**
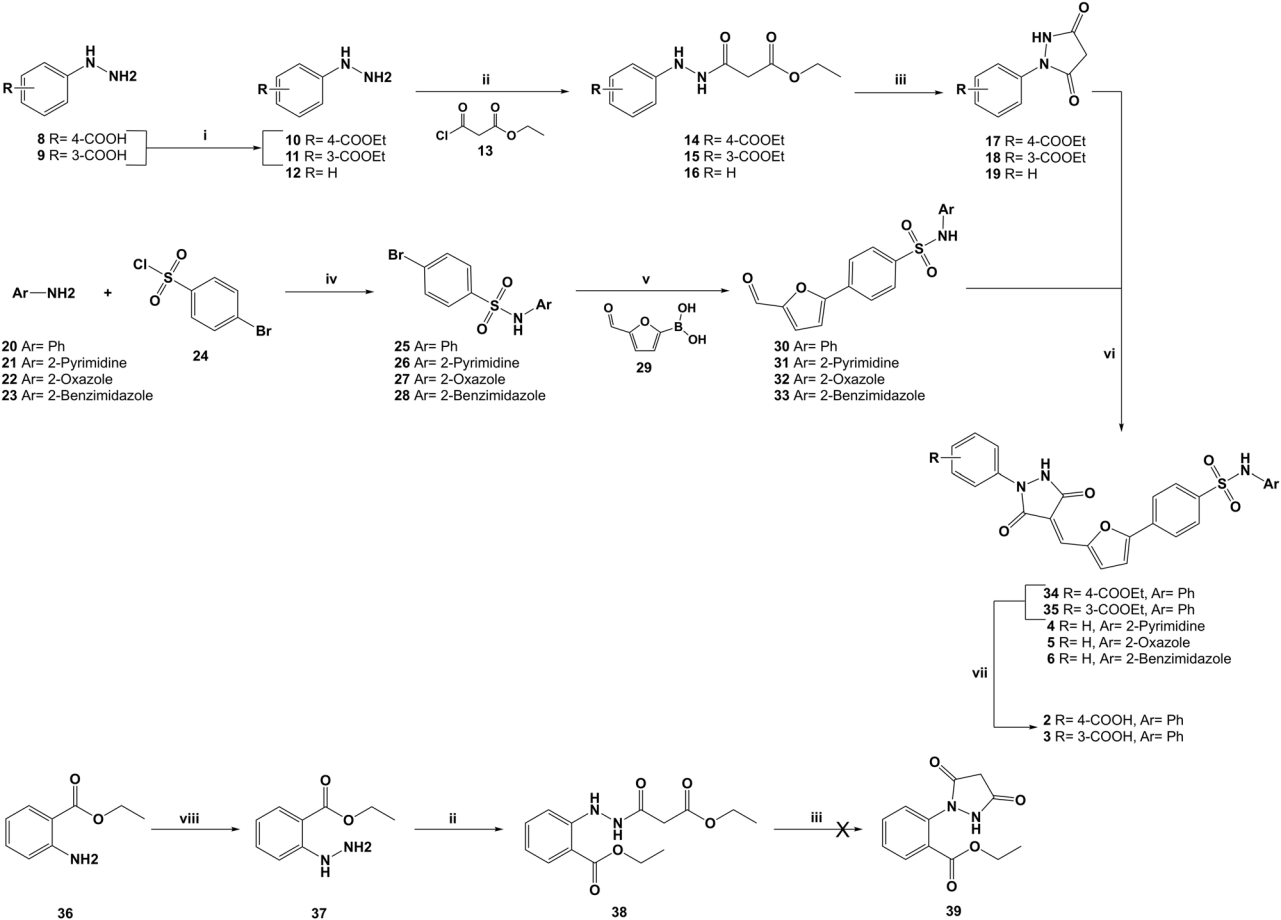
*Reagents and conditions:* (**i**) HCl, EtOH, reflux, o.n., 79–90%; (**ii**) NEt_3_, THF, -10 °C to r.t., 3 h, 75–99%; (**iii**) 1 M NaOH/EtOH, EtOH, r.t., 30 min., 33–55%; (**iv**) Pyridine, r.t., o.n., 68–87%; (**v**) K_3_PO_4_, Pd(dppf)Cl_2_, H_2_O/DMF, MW, 130 °C, 75 min., r.t., o.n. OR Na_2_CO_3_, Pd(OAc)_2_, PPh_3_, H_2_O/DME, MW, 85 °C,10 min., 34–51%; (**vi**) AcOH, 120 °C, o.n., OR EtOH, reflux, o.n., OR MeOH, reflux, 2 h, 48–65%; (**vii**) LiOH, THF/MeOH/H_2_O, r.t., o.n., OR 2 M NaOH, 1,4-Dioxane, o.n., 49–55%; (**viii**) HCl, NaNO_2_, SnCl_2_, 0 °C, 3 h, 99%.

As shown in Fig. 3, differently substituted phenylhydrazines (**10–12**, **37**) were reacted with ethyl-3-chloro-3-oxopropanoate **13** in THF and triethylamine (NEt_3_), giving the desired hydrazides **14–16**, **38**. Ester-susbtituted hydrazines **10–11** were prepared in a good yield by converting the differently substituted hydrazineylbenzoic acids **8–9** into the corresponding ethyl esters via Fisher esterification using hydrochloric acid (HCl) and ethanol (EtOH). An alternative approach was required for the preparation of the *ortho* ethyl ester **37**. In fact, under Fisher reaction conditions, an intramolecular reaction between the carboxylic acid and the hydrazine group occurs, leading to the formation of 3-indanzolinone. The desired ethyl 2-hydrazineylbenzoate **37** was obtained by reacting the ethyl 2-aminobenzoate with sodium nitrite (NaNO_2_) in HCl, and then reducing the intermediate diazonium salt using tin chloride (SnCl_2_). Hydrazides **14–16** were converted into the corresponding 1-arylpyrazolidine-3,5-diones **17–19** through an ester displacement reaction in the presence of sodium hydroxide (NaOH) and EtOH. Unfortunately, the synthesis of *ortho* substituted compound **39** could not be achieved, potentially due to steric hindrance that impedes the cyclization reaction. Treatment of 4-bromobenzene-1-sulfonyl chloride (**24**) with the appropriate aniline (**20–23**) in pyridine produced sulfonamides **25–28**, which were then converted into the aldehyde intermediates **30–33** by Suzuky coupling with (5-formylfuran-2-yl)boronic acid **29**. In particular, for compound **30**, bearing the original phenyl group, our formerly reported conditions using potassium phosphate (K_3_PO_4_) as base, Pd(dppf) as catalyst, water/DMF as solvent and heating under microwave irradiation for 75 min at 130 °C^25^, gave the desired product, whereas no product could be obtained for derivatives **31–33** in these reaction conditions. After exploring various alternative procedures, the best reaction conditions were found using sodium carbonate (Na_2_CO_3_) as base, Pd(OAc)_2_ and PPh_3_ as catalyst, water/DME as solvent, and heating the reaction mixture under microwave irradiation for 10 min at 85 °C, which gave the desired products in moderate yields (34–51%). The two portions were then linked together by reacting aldehydes **30–33** with the appropriate arylpyrazolidine-3,5-dione **17–19** according to a Knoevenagel condensation in acetic acid at 130 °C for 3 h, giving the desired products **5**, **34–35**. The derivatives showing a pyrimidine (**4)** and a 1*H*-benzoimidazole ring (**6**) appeared to degrade under these reaction conditions, therefore **4** was obtained using EtOH under reflux overnight, while **6** was prepared using methanol under reflux for 2 hours. In the last step, hydrolysis of the ethyl ester group was performed for **34** and **35**. The *para* carboxylic acid derivative **2** was obtained by treating the starting ethyl ester with lithium hydroxide (LiOH) overnight at room temperature, while for the *meta* derivative **3** sodium hydroxide (NaOH) and dioxane were used. In fact, using LiOH for the hydrolysis of **35** led to the formation of a hydroxyl amine on the -NH group of the pyrazolidine-3,5-dione ring, with this hydroxylamine side product in a 50:50 ratio with the desired compound **3**. The use of NaOH and dioxane limited significantly the side product formation.

### Synthesis of increased-flexibility analogue 7

**Figure 4:**
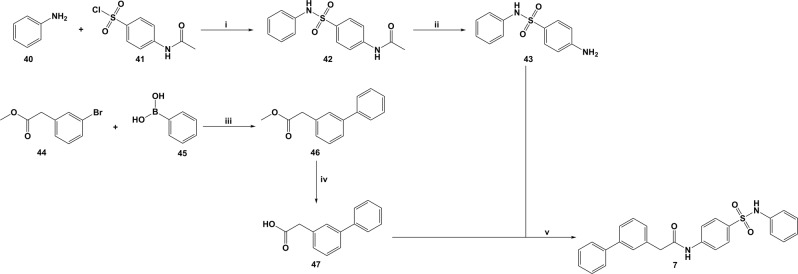
*Reagents and conditions:* (**i**) Pyr, 0 °C, 4 h, 91%; (**ii**) 5 M NaOH, MeOH, 70 °C, 3 h, 89%; (**iii**) Na_2_CO_3_, Pd(PPh_3_)_4_, PhMe/EtOH/H_2_O, 100 °C, 24 h, 75%; (**iv**) LiOH, MeOH/H_2_O, r.t., 2 h, 93%; (**v**) TBTU, DiPEA, DMF, r.t., o.n., 63%.

Compound **7** was prepared in order to initially investigate the influence of the new central *N*,2-diphenylacetamide linker in the RdRp inhibitory activity, in order to preliminarily test the Core Hopping approach. The synthetic pathway reported in Fig. 4 was followed.

Sulfonamide **42** was prepared in almost quantitative yield by reacting aniline **40** and commercially available *N*-acetylsulfanilyl chloride **41** in pyridine at 0 °C. The ester group in **42** was then hydrolysed using NaOH in methanol to afford the free carboxylic acid **43**. A Suzuki coupling reaction, using sodium carbonate (Na_2_CO_3_) as base and tetrakis (Pd[Ph]_3_]_4_) as catalyst, between phenylboronic acid **45** and methyl 2-(3-bromophenyl)acetate **44**, in a solvent mix of toluene/water/ethanol, gave compound **46** in good yield (75%). Hydrolysis of **46** with LiOH in water/methanol gave the free acid **47**, which was then reacted with **43** in dimethylformamide (DMF), *N,N*-diisopropylethylamine (DiPEA) as base and TBTU as coupling agent, to afford the desired final product **7** in moderate yield after flash column chromatography purification.

## Biological Evaluation and Molecular Modelling studies

### Human norovirus RdRp activity inhibition

The newly synthesised compounds, including the carboxylate ester derivatives **34–35** and **7** from the Core Hopping approach, were evaluated for their enzymatic inhibition of human norovirus Sydney 2012 (HuNoV (GII.4)) RdRp activity *in vitro*, using a quantitative fluorescent assay^29^. Compounds were tested at six different concentrations (concentration range examined: 0.1–100 µM) and compared to the relative activity of mock treated samples containing the vehicle only (0.5% DMSO [vol/vol]). PPNDS [Pyridoxal-5′-phosphate-6-(2′-naphthylazo-6′-nitro-4′,8′-disulfonate) tetrasodium salt], a previously reported potent inhibitor of NoV RdRp activity^27^, and **1** were used as positive controls. Dose-dependent inhibitory response curves were used to establish IC_50_ values for the eight test compounds, as reported in Table 1 (see also Supplementary Fig. S1). While IC_50_ values could be attained for six of the eight compounds tested, **7** only reached 20% inhibition at the maximum concentration tested, while **4** reached 15% inhibition at the maximum concentration tested (Supplementary Fig. S2).

**Table 1:** IC_50_ values of hit compounds against HuNoV (GII.4) RdRp activities.

Compound	Structure	IC_50_ values [μM]^A^
**2**	R = 4-COOH, Ar = Ph	17.2 ± 2.4
**3**	R = 3-COOH, Ar = Ph	22.5 ± 6.5
**34**	R = 4-COOEt, Ar = Ph	13.5 ± 5.8
**35**	R = 3-COOEt, Ar = Ph	18.9 ± 3.1
**4**	R = H, Ar = 2-Pyrimidine	n.d.
**5**	R = H, Ar = 2-Oxazole	12.8 ± 2.6
**6**	R = H, Ar = 2-Benzimidazole	26.7 ± 2.0
**7**		n.d.
**1**	R = H, Ar = Ph	5.6^B^
**PPNDS**		1.5 ± 0.25

^A^Mean values ± standard deviations of triplicate datasets are shown from at least three independent experiments.

^B^Activity data as previously reported^29^.

Compounds bearing modifications at different positions of the terminal hydrophobic ring 1 (**2–3**, **34–35**) seem to retain the RdRp inhibitory activity with an IC_50_ in the range of 13–22 µM, slightly higher in comparison with **1** (5.6 µM), but in the same order of magnitude. These results further prove the RdRp inhibitory activity associated with the general scaffold of **1** and suggest that introduction of hydrophilic groups into terminal hydrophobic ring 1 could improve aqueous solubility without disrupting RdRp inhibitory activity. A similar effect is obtained when replacing the terminal hydrophobic ring 2 with an oxazole (**5**) or benzoimidazole (**6**), with an IC_50_ value of 12.8 and 26.7 µM, respectively. On the other hand, the insertion of a pyrimidine ring at this position (**4**) causes loss of activity, with only partial RdRp inhibition seen at 100 µM.

As mentioned above, **7** did not reach 50% inhibition at the test concentrations, showing only 20% RdRp inhibition at 100 µM. This result suggests that compound **1** planarity and rigidity might have an important role for its biochemical activity, as the flexibility conferred by the *N*,2-diphenylacetamide central core almost completely abolishes RdRp inhibition. This effect could be linked with a better overall occupation of the RdRp active site by the active inhibitors, as indicated by molecular docking results shown below.

**Figure 5:**
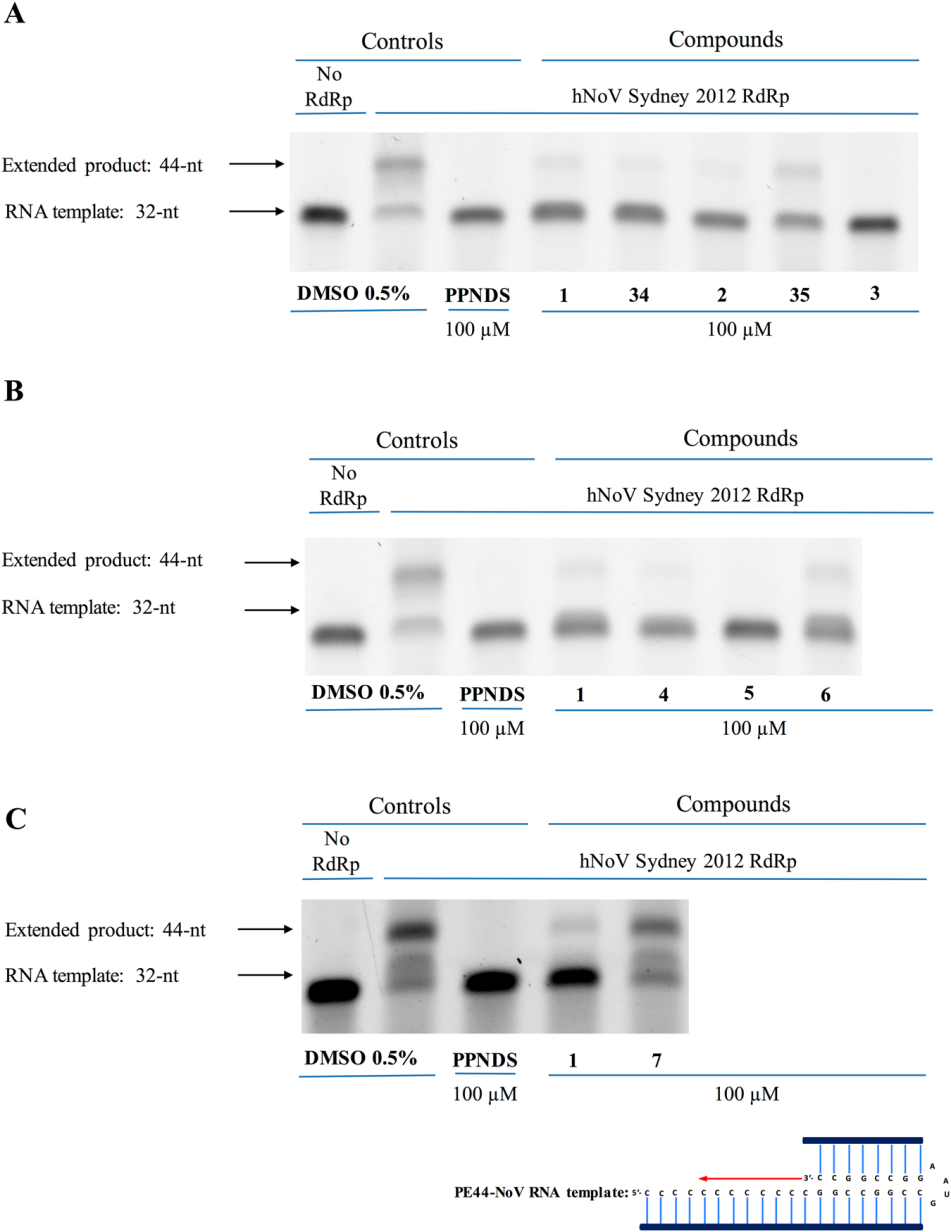
A counter-screen gel-shift assay was used to confirm HuNoV (GII.4) RdRp inhibitory activity of compounds **2**, **3**, **34** and **35** (**A**), **4**, **5**, **6** (**B**) and **7** (**C**). The eight compounds were examined for inhibition of primed elongation activity. PE44-NoV RNA templates (32 nucleotides) were extended (44 nucleotides) by the RdRp in the absence of any test compounds (0.5% DMSO [vol/vol] negative control) or with test compounds at a fixed concentration of 100 μM. PPNDS and compound 1 and were used as positive controls (100 μM) to demonstrate complete inhibition, and no RdRp was used as a negative control. Full-length gels are presented in Supplementary Fig. S3.

To further confirm RdRp inhibition and exclude any potential false positives from the first fluorescence RdRp assay, the eight molecules were then assessed using a gel-shift enzyme activity assay at a fixed concentration of 100 μM, following a procedure previously adopted by our research group^29^. PPNDS was used as positive control and exhibited complete inhibition of transcription at 100 µM (no extension of 32 nucleotide RNA template (PE44-NoV) to 44 nucleotides in the presence of an active RdRp). Seven of the eight compounds exhibited complete or almost complete inhibition of norovirus RdRp activity at 100 μM, comparable to the effect observed for **1**, further confirming their activity as pure polymerase inhibitors (Fig. 5a,b). On the contrary, **7** did not demonstrate inhibition of primed elongation activity in this assay (second higher band on gel shift almost identical to the 0.5% DMSO control, Fig. 5c), confirming that this linker modification abolishes RdRp inhibitory activity.

### Molecular docking studies

**Figure 6:**
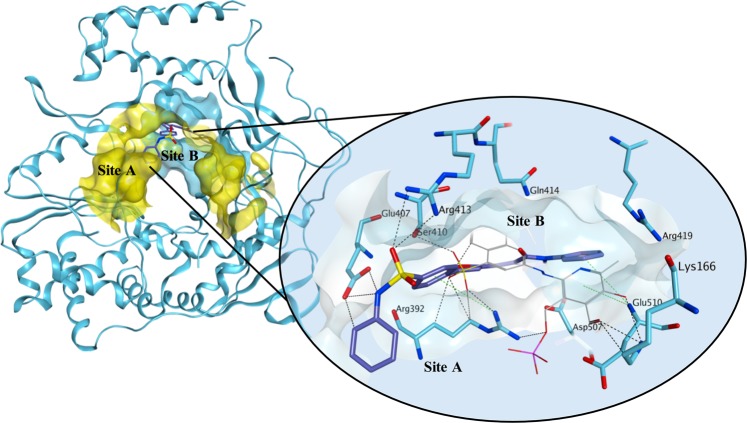
Proposed binding mode for **1** in the human norovirus RdRp. **1** (carbon atoms in lilac) occupies site-B (turquoise surface) and part of site-A (yellow surface). **1** binds in a different orientation compared to PPNDS (carbon atoms in grey), with its phenyl- benzenesulfonamide portion pointing out from the site-B interacting through its sulphonamide group with Glu407 and Arg413 and through an arene-cation interaction between Arg392 and the benzene ring. Arg392 is in the RdRp portion in which site-B and site-A overlap. The phenyl-pyrazolidine portion of **1** occupies the area where PPNDS places its naphthylazo portion, the core area of site-B, making interactions with the surrounding amino acids (Asp507, Glu510). The binding site area is represented as molecular surface. Human norovirus RdRp is represented as turquoise ribbon.

**Figure 7:**
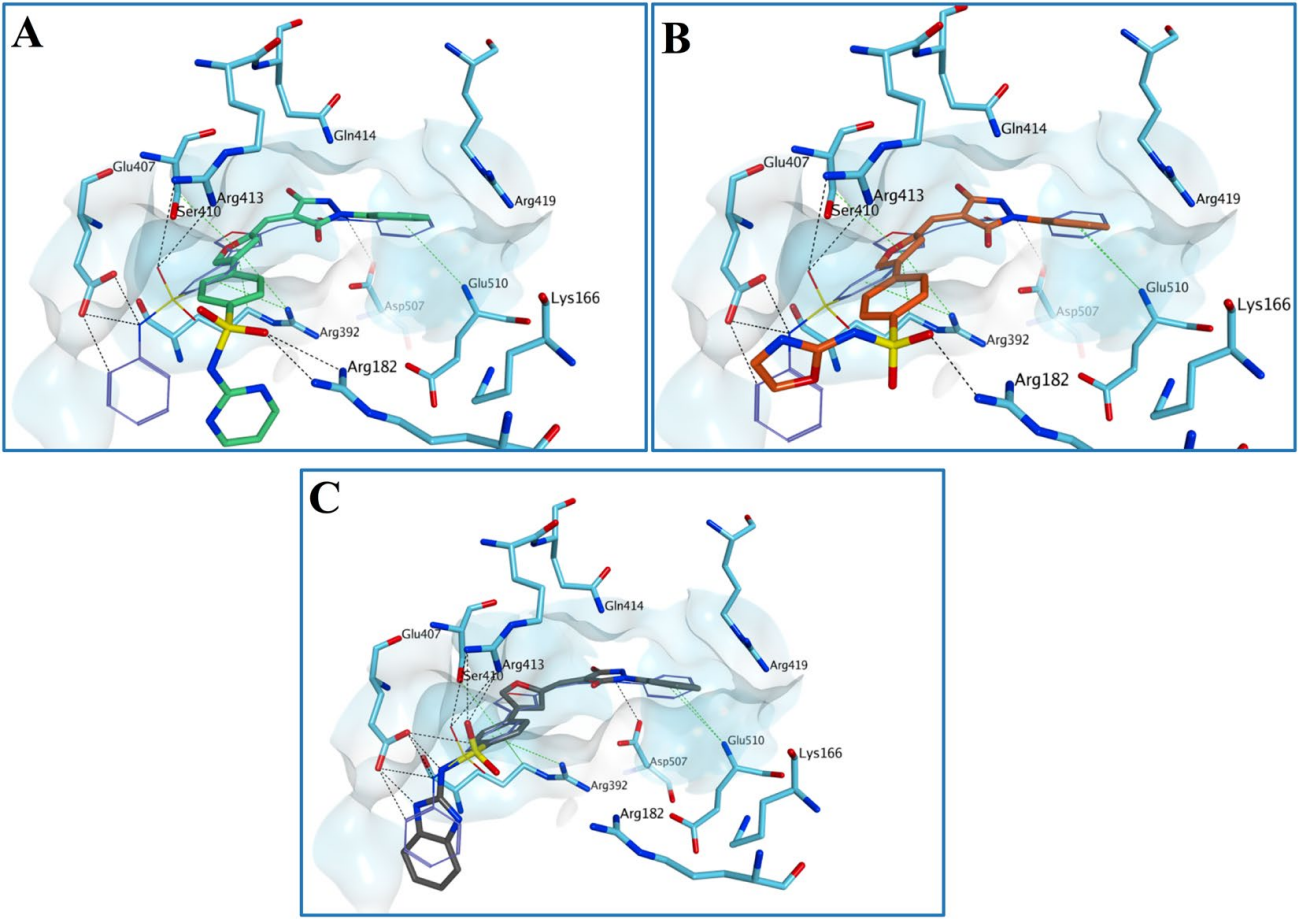
Proposed binding mode for **4** (**A**), **5** (**B**) and **6** (**C**) in the human norovirus RdRp (PDB ID 4LQ3). All the three compounds are predicted to occupy the main nucleic acid binding site of HuNoV RdRp in a similar orientation compared to **1**. The presence of the pyrimidine ring seems to increase the attraction towards Arg182, pushing the compound away from the predicted binding site. The binding site area is represented as molecular surface. Compound **1** binding is reported as comparison (carbon atoms in lilac).

**Figure 8:**
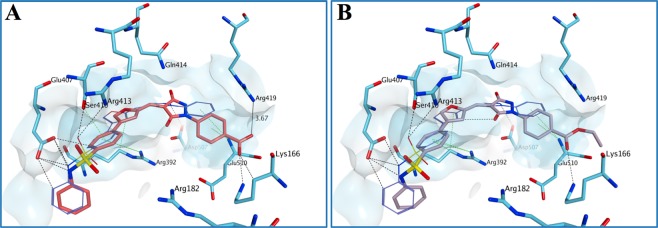
Proposed binding mode for **2** (A) and **34** (**B**) in the human norovirus RdRp (PDB ID 4LQ3). The added carboxylic acid group appears to confer an extra interaction with Lys166, further stabilising the compound **2**-protein complex. This interaction appears to be maintained also in the case of the ester analogue **34**. Moreover, the carboxylic acid group is placed at an optimal distance to interact with Arg419. The binding site area is represented as molecular surface. Compound **1** binding is reported as comparison (carbon atoms in lilac).

**Figure 9:**
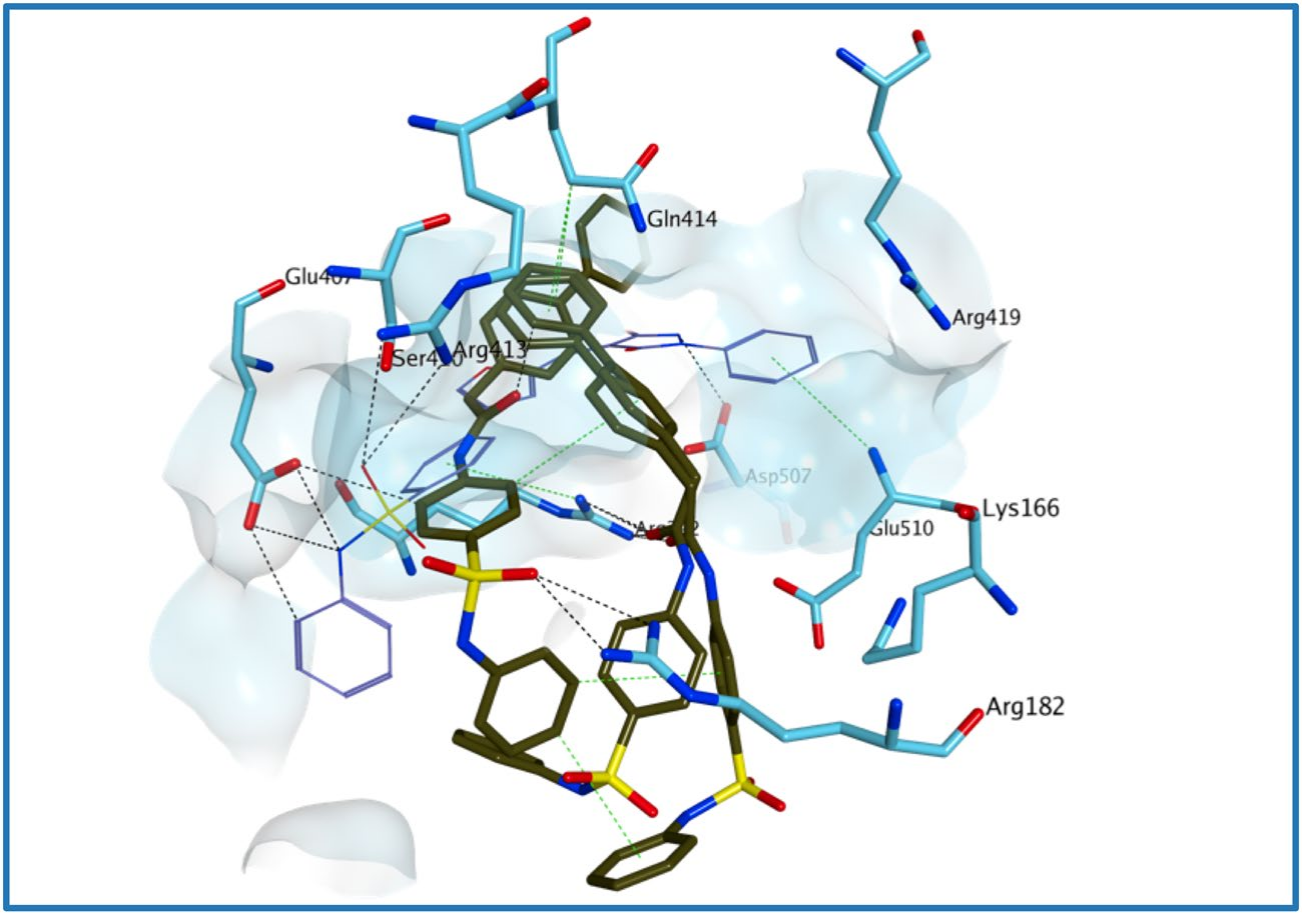
Multiple potential binding modes were obtained for compound **7** in the human norovirus RdRp (PDB ID 4LQ3). The *N*,2-diphenylacetamide central core confers flexibility to the molecule, which assumes a series of conformations that do not allow for an optimal occupation of the binding area. The binding site area is represented as molecular surface. Compound **1** binding is reported as comparison (carbon atoms in lilac).

In our previously published study^29^, both competitive binding studies with PPNDS and molecular docking simulations indicated that **1** likely occupies the central core of site-B and the initial part of site-A of the human norovirus RdRp (Fig. 6). Molecular docking evaluations of the new derivatives on the binding site of **1** in the structure of human norovirus RdRp were performed using Glide SP^33^. Compounds **4–6** are predicted to occupy the main nucleic acid binding site of HuNoV RdRp in a similar orientation compared to **1**, with the *N*-phenyl/heteroaromatic-benzenesulfonamide portion pointing out from site-B towards the RdRp portion in which site-B and site-A overlap (area defined by Arg392), and the differently substituted phenyl-pyrazolidine portion occupying the area where PPNDS places its naphthylazo part (Fig. 7a–c), within the core area of site-B. The added carboxylic group appears to confer an extra interaction with Lys166, further stabilising the ligand-protein complex (Fig. 8a, compound **2**). This interaction appears to be maintained also in the case of the ester analogues **34–35** (Fig. 8b, compound **34**). An interesting observation can be made examining the predicted binding mode of **4**. The presence of the pyrimidine ring seems to increase the attraction towards Arg182, pushing the compound away from the predicted binding site, potentially explaining its reduced enzymatic activity (Fig. 7a). This effect, which is also present at minor extent for **5**, could be a consequence of a different electron/charge delocalisation on the terminal part of the molecule due to the different heteroaromatic rings. Interestingly, **7**, due to the presence of the more flexible *N*,2-diphenylacetamide central core, assumes a series of conformations that do not allow an optimal occupation of the binding area, supporting the observed reduced inhibitory effect of RdRp activity (Fig. 9).

### Cell-based antiviral effect evaluation

The newly prepared compounds **2–7** and **34–35** were evaluated for their antiviral effect against the genogroup V mouse norovirus (MNV) using the mouse macrophage cell line RAW264.7. This assay evaluates the ability of the compounds to protect infected cells from the virus-induced cytopathic effect, thus being suitable to identify inhibition of virus replication at every step of the virus life cycle, and representing a robust cellular assay available for the rapid evaluation of potential norovirus inhibitors. Compounds identified using MNV have shown effects against HuNoV *in vitro* and *in vivo*, as in the case of nucleoside RdRp inhibitor 2′-C-methylcytidine (2CMC)^34–36^. Unfortunately, despite not presenting the aqueous solubility issues we had previously encountered for **1**^29^, no significant antiviral effect could be observed in this assay for the new compounds up to the maximum concentration tested. The new structural modifications, despite retaining inhibition of the RdRp in an isolated enzyme activity assay and showing no cytotoxic effects at the test concentrations in the MNV antiviral assay (Supplementary Table S1), are correlated with a lack of antiviral activity. As these new structures present the same limitation of most non-nucleoside HuNoV polymerase inhibitors reported so far^22^, we were prompted to apply a different computer-aided strategy to further modify the scaffold of **1**, with the aim to achieve an antiviral effect in cellular systems. prompting us to attempt further modifications on their structure.

### Computer-aided flexible alignment approach: new structural modifications on the structure of 1

**Figure 10:**
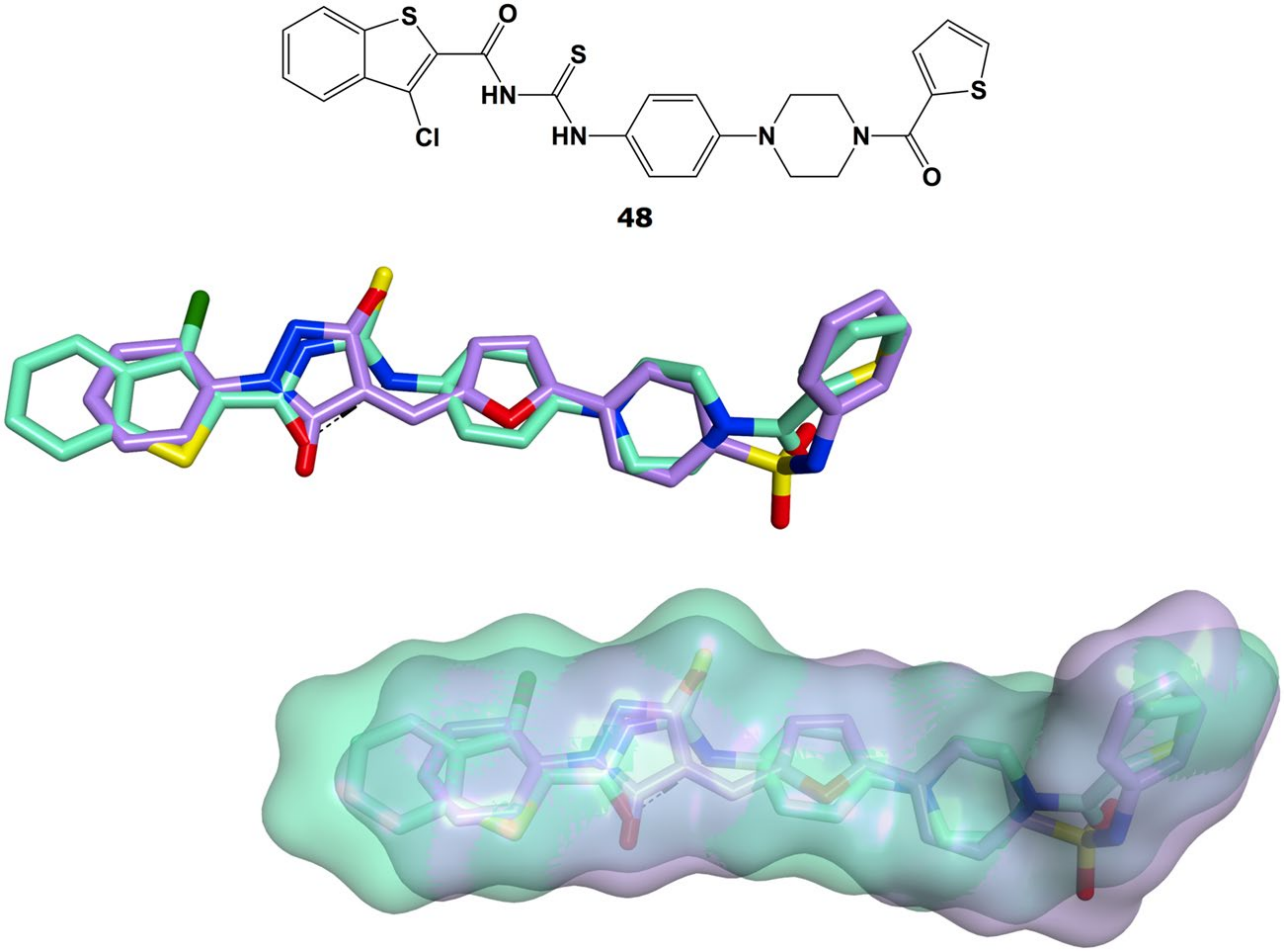
Chemical structure of TPB (**48**, carbon atoms and molecular surface in green) and Flexible Alignment results showing its significant degree of structural and molecular surface overlapping with **1** (carbon atoms and molecular surface in lilac).

Since both attempts at modifying the structure of **1** did not give the desired result of identifying antiviral hits against norovirus replication in cell-based systems, an alternative strategy to identify potential linker replacements for **1** was followed. A conformational search, using MOE conformational search tool^37^, was performed on **1** to identify its lowest energy conformation. This conformation was then kept rigid and used to run a flexible alignment analysis with the Flexible Alignment tool in MOE^37^, in order to search for potential three-dimensional and functional similarities with an in-house small-molecule database of previously reported non-nucleoside inhibitors of viral polymerases, including hepatitis C, Zika and Dengue virus RdRps^38–40^. In particular, the chosen database contains small-molecule compounds reported to have inhibitory activity against viral polymerases, and also to show antiviral effects in cell-based assays. From this study, TPB [3-chloro-N-[({4-[4-(2-thienylcarbonyl)-1-piperazinyl]phenyl}amino)carbonothioyl]-1-benzothiophene-2-carboxamide] (**48**), a recently identified inhibitor of Zika virus RdRp^39^, resulted as the best match for its structural overlapping with **1**, as highlighted in Fig. 10.

**Figure 11:**
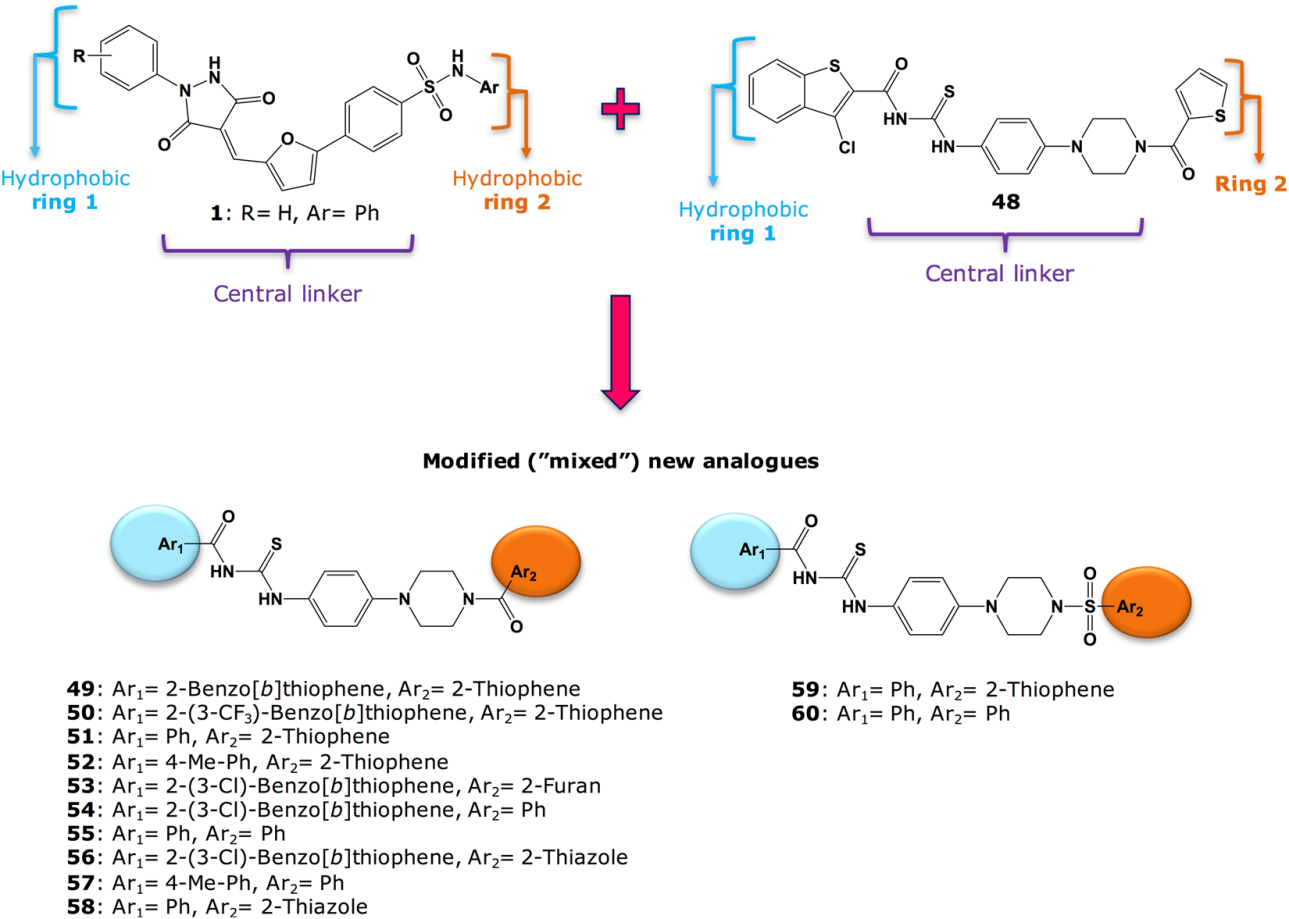
New modifications designed from the structural combination of **1** and **48**.

Evaluation of inhibition of norovirus RdRp activity using an *in vitro* fluorescent, *de novo* RdRp activity assay at a fixed concentration of 20 μM revealed that **48**, which we promptly synthesised in our laboratory, has a mild inhibition of human norovirus RdRp activity (20% inhibition at 20 μM), as reported in the biological evaluation studies section below. This preliminary encouraging result, together with the reported antiviral activity demonstrated for **48** in Vero cells against ZIKV replication^39^, prompted us to explore this scaffold in the search of new norovirus RdRp inhibitors, and most importantly new agents to inhibit HuNoV replication. The structure of **48** was therefore combined with some structural features from **1** and from some of our previously published norovirus RdRp inhibitors^29^, leading to the design of a series of new compounds with a non-planar central linker. These new molecules could present improved solubility and better cell-based antiviral profiles in comparison with **1** and its analogues, as shown in Fig. 11. In addition, in order to verify the activity associated with the scaffold of **48**, a few small structural modifications on its scaffold were also planned and carried out.

## Chemistry

### Synthesis of the newly designed “hybrid” molecules

**Figure 12:**
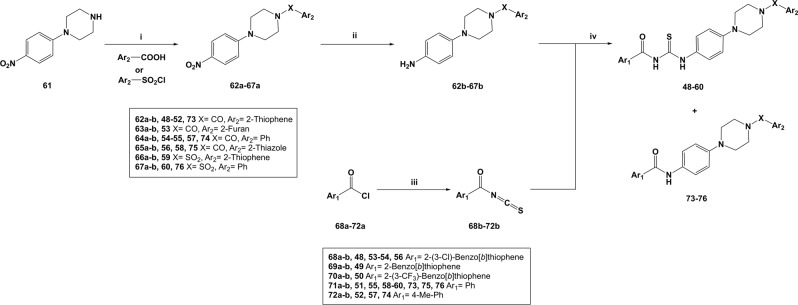
*Reagents and conditions:* (**i**) TBTU, DiPEA, DMF, r.t., 5 h, OR NEt_3_, DCM, 0 °C to r.t., o.n., 81–99%; (**ii**) H_2_, 10% Pd/C, EtOH, r.t., 24 h, 78–99%; (**iii**) CNSH*NH_3_, Me_2_CO, reflux, 1 h, quant.; (**iv**) Me_2_CO, reflux, 2 h, 41–78%.

The 12 newly designed analogues, along with the Flexible Alignment hit **48**, were synthesised according to a four-step synthetic pathway, as shown in Fig. 12.

Briefly, commercial 1-(4-nitrophenyl)piperazine **61** was treated with the appropriately substituted aryl-carboxylic acids or aryl sulfonyl chlorides, to give intermediate nitro-compounds **62a–67a**. In particular, amide-compounds **62a–65a** were obtained through a TBTU-assisted coupling reaction in the presence of DiPEA, in DMF at r.t., while sulfonamide intermediates **66a–67a** were obtained in DCM at 0 °C to r.t., in the presence of NEt_3_. Nitro-intermediates **62a–67a** were then converted into the corresponding substituted aromatic amines **62b–67b** following a catalytic hydrogenation in EtOH, in the presence of wet activated palladium on carbon. Isothiocyanates intermediates **68b–72b** were obtained by treating the corresponding aryl-carbonyl chlorides **68a–72a** with ammonium isothiocyanate, in acetone under reflux conditions. These intermediates were not isolated, but treated *in situ* with the differently substituted aromatic amines **62b–67b**, to give the desired final products **48–60**. In most cases, formation of unwanted amide-byproducts was observed for this reaction, thus explaining the moderate yields obtained in the final step for most of these products. By-products **73–76** were formed in a particularly high amount in the course of the reaction, therefore they were isolated and fully characterised.

## Biological Evaluation and Molecular Modelling Studies

### Evaluation of the inhibitory activity of HuNoV RdRp for analogues 48–60

**Figure 13:**
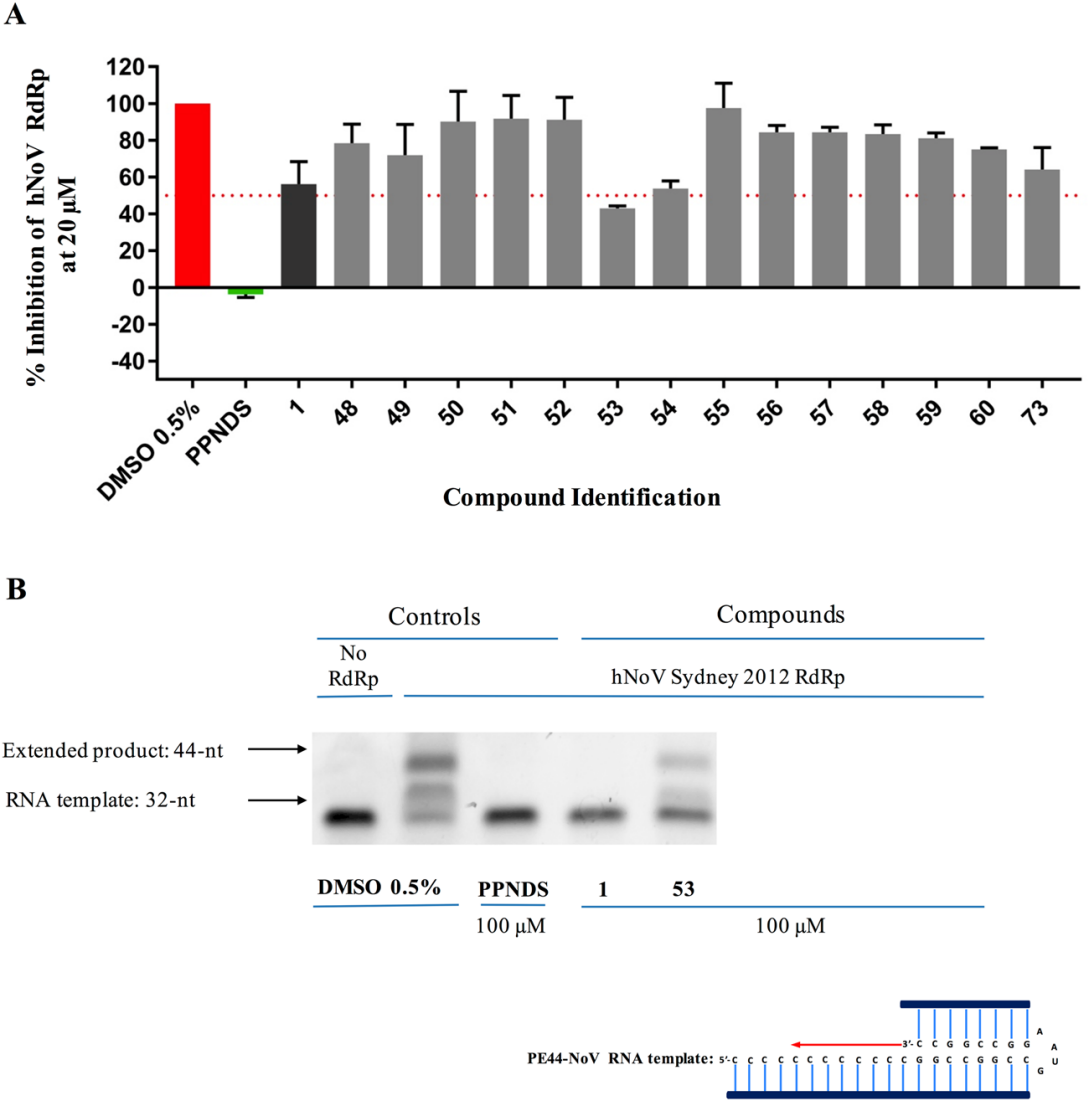
Screening of new derivatives for inhibition of HuNoV (GII.4) RdRp activity. (**A**) The inhibitory effects of 8 compounds were examined against norovirus RdRp activity using a fluorescent activity assay at 20 μM. Compounds **53** and **54** exhibited almost 60% inhibition. PPNDS (green bar) and compound **1** (black bar) were used as positive controls. Percentage of inhibition was normalised to control DMSO (red bar). The mean values of triplicate datasets with standard error of the mean are shown. (**B**) A counter-screen-gel- shift assay was used to confirm norovirus RdRp inhibitory activity of **53**, the most active compound identified in panel (**A**). PE44-NoV RNA templates (32 nucleotides) were extended (44 nucleotides) by the RdRp in the absence of any test compounds (0.5% DMSO [vol/vol] negative control) or with test compounds at a fixed concentration of 100 μM. PPNDS and compound **1** and were used as positive controls (100 μM) to demonstrate complete inhibition, and no RdRp was used as a negative control. Full-length gels are presented in Supplementary Fig. S4.

**Figure 14:**
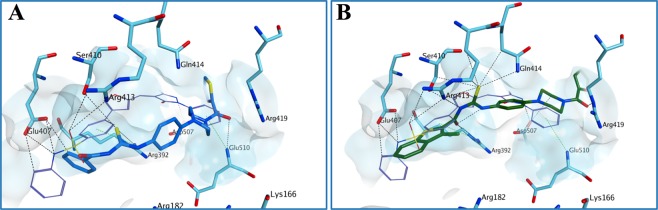
Proposed binding mode for **51** (**A**) and **53** (**B**) to the human norovirus RdRp (PDB ID 4LQ3). Although the sulfur atom of the thiourea forms different H-bonds with the surrounding amino acids, the reduction in central core planarity/rigidity of the new derivatives does not allow an optimal occupation of the HuNoV RdRp active site, potentially reducing the inhibitory activity. The binding site area is represented as molecular surface. Compound **1** binding is reported as comparison (carbon atoms in lilac). Human norovirus RdRp is represented as turquoise ribbon.

The newly prepared compounds **48–60** were initially evaluated against norovirus Sydney 2012 RdRp activity *in vitro* using a quantitative fluorescent assay. Compounds were tested at a fixed concentration of 20 µM and compared to the relative activity of mock treated samples. PPNDS and **1** were used as positive controls. Results of this assay are reported in Fig. 13. Removal of the chlorine atom from position 2 of the benzothiophene ring (**49**) or its replacement with a bigger trifluoromethyl group (**50**) appears not to affect much the RdRp inhibitory activity, with an observed inhibition which is similar to the effect of **48** (15–20% inhibition at 20 µM). A similar effect is obtained for **51** and **52** (~15% inhibition at 20 µM), in which the benzothiophene ring has been replaced with a phenyl ring and a 4-methylphenyl ring respectively. These two rings are structural features present in **1** and in one of its derivatives we have previously reported^25^. Replacement of the thiophene by a furan (**53**) or by an unsubstituted phenyl ring (**54**) increased the degree of HuNoV RdRp inhibition up to 60% at 20 µM, whereas introduction of a thiazole ring (**56**) does not affect enzymatic inhibition activity. From these observations, the thiophene ring appears not to influence inhibitory activity. Derivatives in which the central core of **48** has been used to link the two terminal hydrophobic rings of **1** or of its derivatives we have previously reported^25^ (**55**, **57**, **58**) appear to retain the RdRp inhibitory activity found for **48** (~20% at 20 µM). A similar effect is obtained when a sulfonyl group, as in our hit **1**, is used to link ring 2, regardless if it is a thiazole (**59**) or an unsubstituted phenyl ring (**60**), to the rest of the molecule. Interestingly, **73**, one of the amide side products formed during the last synthetic step for this series of compounds, reduced the polymerase activity of ~40% at 20 µM. **53**, the most active compound found in this series, also exhibits a significant inhibition of HuNoV RdRp activity at 100 µM in the gel-shift assay, confirming the polymerase inhibitory function of the new scaffold. Overall, even if showing a reduced biochemical activity in comparison with **1**, some of the new derivatives can be considered as modest HuNoV RdRp inhibitors at 20 µM. This reduced activity in comparison with **1** could to some extent have been expected, since, as seen for **7**, the reduction in central core planarity/rigidity in the new derivatives would likely reduce their interactions and optimal fitting of the HuNoV RdRp active site. This hypothesis is in line with molecular docking results obtained for **51** and **53**, as shown in Fig. 14.

### Cell-based antiviral effect evaluation

Since the main scope of this work was to find new norovirus inhibitors which show antiviral effects against both the HuNoV RdRp and in norovirus-infected cells, priority was given to the evaluation of most of the new molecules for their antiviral activity against MNV in infected RAW cells. The nucleoside 2′-C-methylcytidine was used as positive control. The test compounds were evaluated at eight different concentrations (range 0.6–100 µM) and their ability to reduce the virus-induced cytopathic effect was assessed.

Five out of 10 compounds tested were found to have an interesting antiviral EC_50_ in the 20–100 µM range in this assay, as reported in Table 2. These new derivatives showed a slightly better EC_50_ if compared with what we had previously found for **1**^29^, but more interestingly they were not cytotoxic at the test concentrations, whereas **1** exhibited a mean half maximal cytotoxic concentration (CC_50_) of 62.8 μM in a similar MNV cell-based assay^29^.

**Table 2:** EC_50_ and CC_50_ values of **48–60** in the MNV CPE reduction assay.

Compound	Structure	EC_50_ values [μM]^A^	CC_50_ values [μM]^A^
**48 (TPB)**	Ar_1_ = 2-(3-Cl)-Benzo[b]thiophene, X = CO, Ar_2_ = 2-Thiophene	**58.2 ± 20.7**	**>100**
**49**	Ar_1_ = 2-Benzo[b]thiophene, X = CO, Ar_2_ = 2-Thiophene	>100	>100
**50**	Ar_1_ = 2-(3-CF_3_)-Benzo[b]thiophene, X = CO, Ar_2_ = 2-Thiophene	n.d.^B^	n.d.^B^
**51**	Ar_1_ = Ph, X = CO, Ar_2_ = 2-Thiophene	n.d.^B^	n.d.^B^
**52**	Ar_1_ = 4-Me-Ph, X = CO, Ar_2_ = 2-Thiophene	**45.1 ± 10.8**	**>100**
**53**	Ar_1_ = 2-(3-Cl)-Benzo[b]thiophene, X = CO, Ar_2_ = 2-Furan	>100	9.4 ± 2.3
**54**	Ar_1_ = 2-(3-Cl)-Benzo[b]thiophene, X = CO, Ar_2_ = Ph	>100	4.5 ± 0.7
**55**	Ar_1_ = Ph, X = CO, Ar_2_ = Ph	>100	61.9
**56**	Ar_1_ = 2-(3-Cl)-Benzo[b]thiophene, X = CO, Ar_2_ = 2-Thiazole	**66.1 ± 12.3**	**>100**
**57**	Ar_1_ = 4-Me-Ph, X = CO, Ar_2_ = Ph	>100	>100
**58**	Ar_1_ = Ph, X = CO, Ar_2_ = 2-Thiazole	n.d.	n.d.
**59**	Ar_1_ = Ph, X = SO_2_, Ar_2_ = 2-Thiophene	**43.8 ± 16.9**	**>100**
**60**	Ar_1_ = Ph, X = SO_2_, Ar_2_ = Ph	**66.6** ± **12.3**	**>100**
**2CMC**		2.0 ± 1.0	16 ± 2

^A^The mean values ± standard deviations are shown from at least three independent experiments.

^B^Not determined.

As a further step, the five hit compounds which showed activity in the MNV assay were tested against HuNoV using a replicon system, i.e. a human gastric tumor-1 (HGT-1) cell line which stably expresses the genome of a HuNoV GI.1 virus^41^. Given that in this system the gene encoding for the major capsid protein is replaced by a neomycin resistance gene, no new virus particles will be produced but the non-structural proteins are expressed and the replication of the genomic RNA can be studied^42^.

While **48** and **56** do not display any significant antiviral activity in this second assay, **52**, **59** and **60** inhibit the viral replication of HuNoV GI with EC_50_ values in the low micromolar range, as shown in Table 3. The lack of activity encountered for **48** and **56** in this assay could be a consequence of several factors, such as differences on the envelope and capsid proteins between MNV and HuNoV, which could affect the ability of the two compounds to reach the viral RdRp, or different readouts and sensitivity of the two assays in detecting the inhibitory activity of the compounds.

**Table 3:** EC_50_ values found for the six tested compounds the HuNoV GI replicon assay.

Compound	Structure	M [μM]^A^
48 (TPB)	Ar_1_ = 2-(3-Cl)-Benzo[b]thiophene, X = CO, Ar_2_ = 2-Thiophene	>100
52	Ar_1_ = 4-Me-Ph, X = CO, Ar_2_ = 2-Thiophene	**6.1 ± 3.9**
56	Ar_1_ = 2-(3-Cl)-Benzo[b]thiophene, X = CO, Ar_2_ = 2-Thiazole	>100
59	Ar_1_ = Ph, X = SO_2_, Ar_2_ = 2-Thiophene	**10.9 ± 11.1**
60	Ar_1_ = Ph, X = SO_2_, Ar_2_ = Ph	8.5 ± 2.1
Rupintrivir^42^		1.5 ± 0.4

^A^The mean values ± standard deviations of triplicate datasets are shown from at least three independent experiments.

Even if showing an interesting EC_50_ of 8 µM, **60** was found to form crystals starting from a 25 µM concentration, thus indicating that its solubility should still be improved. **52** and **59** display instead the most interesting antiviral activity profile found so far, with EC_50_ values against HuNoV GI replication of 6 µM and 10 µM, respectively, providing a new antiviral scaffold for norovirus which shows promise for additional investigations. Overall, due to their low-micromolar inhibitory activity against norovirus replication *in vitro*, **52** and **59** represent one of the few successful examples of non-nucleoside HuNoV polymerase inhibitors which also show antiviral activity against the viral replication in a cell-based system. These compounds represent a promising starting point for further structural optimisation and SAR evaluations. In particular, current research efforts are now ongoing to further improve the drug-like properties of these compounds, and to increase their potency in both biochemical and cellular assays.

## Conclusions and Future Work

Starting from a rational approach to modify the structure of one HuNoV RdRp inhibitor, **1**, which was recently identified in our research group, we have explored different modifications on its scaffold, in order to increase its drug-like potential as an antiviral agent. Despite the fact that most of the newly modified compounds retain inhibitory activity on the viral polymerase, none of them resulted as an antiviral hit when tested in a MNV CPE reduction assay. In an attempt to further modify the scaffold of **1** and achieve an antiviral effect in cellular systems, we have performed a computer-aided approach in order to identify potential similarities between **1** and previously reported non-nucleoside inhibitors of viral polymerases, which also display good antiviral activities against the replication of different viruses in cell-based assays. Following this rationale, we have recognised significant structural similarities between our biochemical hit **1** and a recently reported inhibitor of Zika virus polymerase, **48**. Rationally combining together different structural features of **1** and **48**, we have designed and synthesised a new family of 12 modified analogues. Among them, different displayed at least partial inhibition of HuNoV RdRp activity in a quantitative fluorescent assay. Most interestingly, five of these compounds were found to reduce MNV-induced cytopathic effect with EC_50_ values in the micromolar range, without being associated with significant cytotoxicity. These five hits were then evaluated in a human norovirus replicon assay harbouring a genogroup GI, and two of them, **52** and **59**, were found to inhibit HuNoV replication with EC_50_ values in the low micromolar range, revealing a new antiviral scaffold for human norovirus. These compounds represent one of the few examples of non-nucleoside polymerase inhibitors also showing significant antiviral effect against human norovirus replication in a cell-based system. Based on these findings, additional investigations on these new structures are currently ongoing to further explore their potential as antiviral agents. In particular, further structural optimisation is the main focus of present research efforts, which aim to identify suitable preclinical antiviral candidates for the treatment of norovirus infections.

## Experimental

### Chemistry

All solvents and reagents were used as obtained from commercial sources unless otherwise indicated. All solvents used for chromatography were HPLC grade from Fisher Scientific (UK). All reactions were performed under a nitrogen atmosphere^29^. ^1^H and ^13^C-NMR spectra were recorded with a Bruker Avance III HD spectrometer operating at 500 MHz for ^1^H and 125 MHz for ^13^C, with Me_4_Si as internal standard. Deuterated chloroform was used as the solvent for NMR experiments, unless otherwise stated. ^1^H chemical shifts values (δ) are referenced to the residual non-deuterated components of the NMR solvents (δ  = 7.26 ppm for CHCl_3_, etc.)^29^. The ^13^C chemical shifts (δ) are referenced to CDCl_3_ (central peak, δ = 77.0 ppm). TLC was performed on silica gel 60 F254 plastic sheets. Flash column chromatography was performed using silica an Isolera Biotage system. UPLC-MS analysis was conducted on a Waters UPLC system with both Diode Array detection and Electrospray (+′ve and −′ve ion) MS detection. The stationary phase was a Waters Acquity UPLC BEH C18 1.7 um 2.1 × 50 mm column. The mobile phase was LC-MS grade H_2_O containing 0.1% formic acid (A) and LC-MS grade MeCN containing 0.1% formic acid (B). Column temperature: 40 °C. Sample diluent: MeCN. Sample concentration 1 µg/mL. Injection volume 2 µL^29^. Three alternative methods were used:

Linear gradient standard method (A): 90% A (0.1 min), 90–0% A (2.5 min), 0% A (0.3 min), 90% A (0.1 min); flow rate 0.5 mL/min.

Linear gradient standard method (B): 90% A (0.1 min), 90–0% A (2.1 min), 0% A (0.8 min), 90% A (0.1 min); flow rate 0.5 mL/min.

Linear gradient standard method (C): 90% A (0.1 min), 90–0% A (1.5 min), 0% A (1.4 min), 90% A (0.1 min); flow rate 0.5 mL/min.

All compounds tested in biological assays were>95% pure. Purity of intermediates was>90%, unless otherwise stated. All intermediates were generally prepared according to literature procedures, which are described in detail along with compound characterisation in the Supporting Information. Details for the preparation and full characterisation of the new target final compounds are given below.

### General method for the preparation of *N*-aryl-4-(5-((3,5-dioxo-1-phenylpyrazolidin-4-ylidene)methyl)furan-2-yl)benzenesulfonamides 5, 34–35

The appropriately substituted 1-phenylpyrazolidine-3,5-dione **17–19** (1 mmol) and the differently substituted *N*-(aryl)-(5-formylfuran-2-yl)benzenesulfonamides **30**, **32** (1 mmol) were suspended in AcOH (15 ml/mmol) and the mixture was stirred at 120 °C for 3 hours. The mixture was then cooled to room temperature, quenched with water and the precipitate obtained was filtered, washed with water and dried under vacuum. The crude product was purified by trituration to afford the title compound.

### (*E*)-4-(5-((3,5-dioxo-1-phenylpyrazolidin-4-ylidene)methyl)furan-2-yl)-*N*-(oxazol-2-yl)benzenesulfonamide (5)

Purified by trituration from DCM. Obtained in 65% yield as a light brown solid. ^1^H-NMR (DMSO-d_6_), δ: 12.25 (bs, 1 H), 11.19 (bs, 1 H), 8.54–8.50 (m, 1 H), 8.14–8.11 (m, 2 H), 7.99–7.96 (m, 2 H), 7.77–7.70 (m, 3 H), 7.64–7.63 (m, 1 H), 7.59–7.58 (m, 1 H), 7.47–7.43 (m, 2 H), 7.32–7.30 (m, 1 H), 7.24–7.18 (m, 1 H). ^13^C-NMR (DMSO-d_6_), δ: 158.5, 158.4, 156.7, 150.7, 150.6, 144.1, 131.7, 129.4, 129.3, 129.2, 127.4, 127.3, 125.8, 118.7, 116.3, 115.1, 113.7, 113.4, 113.3. UPLC-MS (Method C): R_t_ 1.84 min, MS [ESI, m/z]: 477.1 [M + H].

Anal. Calcd for C_23_H_16_N_4_O_6_S: C, 57.98; H, 3.38; N, 11.76. Found: C, 57.69; H, 3.12; N, 11.95.

### Ethyl(*E*)-4-(3,5-dioxo-4-((5-(4-(*N*-phenylsulfamoyl)phenyl)furan-2-yl)methylene)pyrazolidin-1-yl)benzoate (34)

Purified by trituration from DCM. Obtained in 48% yield as a light brown solid. ^1^H-NMR (DMSO-d_6_), δ: 11.53 (bs, 1 H), 10.36 (bs, 1 H), 8.56–8.50 (m, 1 H), 8.11 (d, J = 8.4 Hz, 2 H), 8.03 (d, J = 8.6 Hz, 2 H), 7.96–7.90 (m, 2 H), 7.87–7.84 (m, 2 H), 7.70–7.65 (m, 1 H), 7.58–7.56 (m, 1 H), 7.26–7.23 (m, 2 H), 7.11 (d, J = 8.6 Hz, 2 H), 7.06–7.03 (m, 1 H), 4.31 (q, J = 7.1 Hz, 2 H), 1.33 (t, J = 7.1 Hz, 3 H). ^13^C-NMR (DMSO-d_6_), δ: 175.9, 165.7, 165.6, 158.1, 158.0, 150.8, 140.3, 138.8, 132.5, 130.7, 130.6, 129.7, 128.1, 128.0, 126.1, 124.9, 120.9, 120.8, 115.1, 114.9, 113.9, 61.1, 14.7. UPLC-MS (Method C): R_t_ 2.30 min, MS [ESI, m/z]: 558.1 [M + H]. Anal. Calcd for C_29_H_23_N_3_O_7_S: C, 62.47; H, 4.16; N, 7.54. Found: C, 62.61; H, 3.97; N, 7.39.

### Ethyl(*E*)-3-(3,5-dioxo-4-((5-(4-(N-phenylsulfamoyl)phenyl)furan-2-yl)methylene)pyrazolidin-1-yl)benzoate (35)

Purified by trituration from DCM. Obtained in 58% yield as a light brown solid. ^1^H-NMR (DMSO-d_6_), δ: 11.28 (bs, 1 H), 10.35 (bs, 1 H), 8.51–8.39 (m, 2 H), 8.10 (d, J = 8.5 Hz, 2 H), 8.01–7.97 (m, 1 H), 7.85 (d, J = 8.5 Hz, 2 H), 7.78–7.76 (m, 1 H), 7.71–7.68 (m, 1 H), 7.61–7.55 (m, 2 H), 7.26–7.23 (m, 2 H), 7.12–7.10 (m, 2 H), 7.06–7.03 (m, 1 H), 4.35 (q, J = 7.1 Hz, 2 H), 1.34 (t, J = 7.1 Hz, 3 H). ^13^C-NMR (DMSO-d_6_), δ: 176.6, 165.9, 165.8, 150.8, 140.3, 140.2, 137.8, 132.5, 131.1, 131.0, 129.9, 129.8, 129.7, 128.2, 128.1, 126.1, 124.8, 120.9, 120.8, 115.1, 113.9, 113.8, 61.5, 14.6. UPLC-MS (Method C): R_t_ 2.32 min, MS [ESI, m/z]: 558.1 [M + H].

Anal. Calcd for C_29_H_23_N_3_O_7_S: C, 62.47; H, 4.16; N, 7.54. Found: C, 62.32; H, 4.24; N, 7.39.

### Preparation of (*E*)-4-(5-((3,5-dioxo-1-phenylpyrazolidin-4-ylidene)methyl)furan-2-yl)-*N*-(pyrimidin-2-yl)benzenesulfonamide (4)

To a suspension of 1-phenylpyrazolidine-3,5-dione **19** (0.02 g, 0.13 mmol) in EtOH (10 ml/mmol) were added 4-(5-formylfuran-2-yl)-*N*-(pyrimidin-2-yl)benzenesulfonamide **31** (0.05, 0.15 mmol) and one drop of pyridine. The reaction was stirred under reflux overnight, then cooled to room temperature and the precipitate formed was filtered off and washed with cold EtOH and dried under vacuum. The crude product was purified by automated flash column chromatography (Biotage Isolera One, SNAP KP Sil 10 g) eluting with DCN:MeOH:Net_3_ 100:0:0 v/v increasing to 80:15:5 v/v in 15 CV. Obtained in 51% yield as a light brown solid. ^1^H-NMR (DMSO-d_6_), δ: 11.90 (bs, 2 H), 8.54–8.47 (m, 3 H), 8.16–8.13 (m, 2 H), 8.10–8.08 (m, 2 H), 7.79–7.76 (m, 3 H), 7.59–7.58 (m, 1 H), 7.48–7.44 (m, 2 H), 7.23–7.19 (m, 1 H), 7.08–7.04 (m, 1 H). ^13^C-NMR (DMSO-d_6_), δ: 158.1, 157.3, 157.2, 150.8, 150.7, 132.5, 129.4, 129.3, 128.9, 128.3, 125.7, 125.2, 119.1, 118.8, 117.2, 115.5, 115.3, 113.8, 113.7, 113.0. UPLC-MS (Method C): R_t_ 1.94 min, MS [ESI, m/z]: 488.1 [M + H].

Anal. Calcd for C_24_H_17_N_5_O_5_S: C, 59.13; H, 3.52; N, 14.37. Found: C, 59.28; H, 3.40; N, 14.51.

### Preparation of (*E*)-*N*-(1H-benzo[d]imidazol-2-yl)-4-(5-((3,5-dioxo-1-phenylpyrazolidin-4-ylidene) methyl)furan-2-yl)benzenesulfonamide (6)

To a solution of 1-phenylpyrazolidine-3,5-dione **19** (0.19 mmol) in MeOH (10 ml/mmol) was added *N*-(1H-benzo[d]imidazol-2-yl)-4-(5-formylfuran-2-yl)benzenesulfonamide **33** (0.19 mmol). The reaction was stirred at reflux for 2 hours. The precipitate formed was filtered off, washed with cold MeOH and dried under vacuum. The crude product was purified by automated flash column chromatography (Biotage Isolera One, SNAP KP Sil 10 g) eluting with DCN:MeOH 100:0:0 v/v increasing to 90:10 v/v in 10 CV. Obtained in 55% yield as a light brown solid. ^1^H-NMR (DMSO-d_6_), δ: 11.38 (bs, 1 H), 8.43–8.35 (m, 1 H), 8.19–8.15 (m, 4 H), 7.79–7.73 (m, 2 H), 7.72–7.70 (m, 1 H), 7.68–7.63 (m, 1 H), 7.62–7.60 (m, 1 H), 7.46–7.43 (m, 2 H), 7.21–7.18 (m, 3 H), 7.17–7.14 (m, 2 H), 7.06–7.03 (m, 1 H). ^13^C-NMR (DMSO-d6), δ: 157.1, 152.5, 151.2, 151.1, 143.3, 136.8, 136.7, 134.6, 130.4, 129.4, 129.3, 128.3, 128.2, 126.5, 126.4, 125.3, 121.1, 116.5, 116.1, 114.7, 114.6, 114.1, 112.7. UPLC-MS (Method C): R_t_ 1.93 min, MS [ESI, m/z]: 526.2 [M + H]. Anal. Calcd for C_27_H_19_N_5_O_5_S: C, 61.71; H, 3.64; N, 13.33. Found: C, 61.54; H, 3.52; N, 13.55.

### Preparation of (*E*)-4-(3,5-dioxo-4-((5-(4-(*N*-phenylsulfamoyl)phenyl)furan-2-yl)methylene) pyrazolidin-1-yl)benzoic acid (2)

To a solution of ethyl (*E*)-4-(3,5-dioxo-4-((5-(4-(*N*-phenylsulfamoyl)phenyl)furan-2-yl)methylene)pyrazolidin-1-yl) benzoate **34** (0.18 mmol) in 18 ml of THF:MeOH:H_2_O (4:1:1) was added LiOH (0.53 mmol). The reaction was stirred at room temperature overnight. The mixture was acidified with 1 N HCl aqueous solution. The solvent was reduced in volume to remove MeOH and THF, and the aqueous layer was extracted with EtOAc (3 × 20 ml). The organic layer was dried over MgSO_4_ and concentrated under vacuum. The crude product was purified by trituration from DCM. Obtained in 58% yield as a light brown solid. ^1^H-NMR (DMSO-d_6_), δ: 13.05 (bs, 1 H), 11.88 (bs, 1 H), 10.27 (s, 1 H), 8.07 (d, J = 8.7 Hz, 2 H), 7.90–7.88 (m, 4 H), 7.81 (d, J = 8.7 Hz, 2 H), 7.30 (d, J = 3.6 Hz, 1 H), 7.26–7.22 (m, 2 H), 7.16 (d, J = 3.6 Hz, 1 H), 7.12–7.09 (m, 2 H), 7.05–7.02 (m, 1 H). ^13^C-NMR (DMSO-d_6_), δ: 167.1, 155.6, 151.8, 150.9, 146.2, 144.9, 138.3, 138.0, 134.0, 130.1, 129.8, 129.6, 128.0, 125.3, 124.7, 124.2, 120.9, 120.8, 117.1, 110.9, 103.6. UPLC-MS (Method C): R_t_ 2.04 min, MS [ESI, m/z]: 528.1 [M-H]. Anal. Calcd for C_27_H_19_N_3_O_7_S: C, 61.24; H, 3.62; N, 7.94. Found: C, 61.50; H, 3.51; N, 8.03.

### Preparation of (*E*)-3-(3,5-dioxo-4-((5-(4-(*N*-phenylsulfamoyl)phenyl)furan-2-yl)methylene) pyrazolidin-1-yl)benzoic acid (3)

Ethyl (*E*)-3-(3,5-dioxo-4-((5-(4-(*N*-phenylsulfamoyl)phenyl)furan-2-yl)methylene)pyrazolidin-1-yl)benzoate **35** (0.07 mmol) was added to a mixture of 2 M NaOH aqueous solution (1 ml) and dioxane (1 ml). The reaction was stirred vigorously at room temperature overnight. The solvent was reduced in volume under vacuum and acidified by addition of 1 M HCl solution. The water layer was then extracted with EtOAc (3 × 20 ml). The organic portions were combined, dried over MgSO_4_ and concentrated under vacuum. The crude product was purified by automated flash column chromatography (Biotage Isolera One, SNAP KP Sil 10 g) eluting with DCM:MeOH 100:0:0 v/v increasing to 80:20 v/v in 15 CV. Obtained in 49% yield as a light brown solid. ^1^H-NMR (DMSO-d_6_), δ: 13.04 (bs, 2 H), 10.35 (bs, 1 H), 8.54–8.50 (m, 1 H), 8.43–8.39 (m, 1 H), 8.12–8.09 (m, 2 H), 8.05–7.99 (m, 1 H), 7.86–7.84 (m, 2 H), 7.75–7.73 (m, 1 H), 7.67–7.63 (m, 1 H), 7.58–7.55 (m, 2 H), 7.26–7.22 (m, 2 H), 7.12–7.10 (m, 2 H), 7.06–7.03 (m, 1 H). ^13^C-NMR (DMSO-d_6_), δ: 167.4, 156.2, 151.6, 150.8, 146.3, 145.5, 140.2, 137.8, 132.6, 129.6, 129.5, 128.0, 125.9, 124.8, 124.3, 120.9, 120.3, 117.4, 109.9, 103.4. UPLC-MS (Method C): R_t_ 2.04 min, MS [ESI, m/z]: 528.1 [M-H]. Anal. calcd for C_27_H_19_N_3_O_7_S: C, 61.24; H, 3.62; N, 7.94. Found: C, 61.01; H, 3.43; N, 8.06.

### Preparation of 2-([1,1′-biphenyl]-3-yl)-*N*-(4-(N-phenylsulfamoyl)phenyl)acetamide (7)

DiPEA (1.1 mmol) was added to a solution of 2-([1,1′-biphenyl]-3-yl)acetic acid **47** (0.44 mmol), 4-amino-*N*-phenylbenzenesulfonamide **43** (0.48 mmol) and TBTU (0.48 mmol) in 3 mL of anhydrous DMF under N_2_ atmosphere at room temperature. The reaction was stirred at r.t. over night, then diluted with EtOAc (20 mL) and washed with saturated aqueous NH_4_Cl solution (15 mL), saturated aqueous NaHCO_3_ solution (15 mL) and brine (10 mL). The organic phase was dried over MgSO_4_ and concentrated under vacuum.

The crude product was purified by automated flash column chromatography (Biotage Isolera One, SNAP KP Sil 10 g) eluting with *n*-hexane:EtOAc 100:0 v/v increasing to 0:100 v/v in 12 CV. Obtained in 63% yield as an off-white solid. ^1^H-NMR (DMSO-d6), δ: 3.75 (s, 2 H), 6.98–7.02 (m, 1 H), 7.06 (d, J = 8.2 Hz, 2 H), 7.19–7.22 (m, 2 H), 7.30–7.32 (m, 1 H), 7.35–7.43 (m, 2 H), 7.46–7.49 (m, 2 H), 7.54 (d, J = 7.1 Hz, 1 H), 7.62–7.65 (m, 3 H), 7.68 (d, J = 8.8 Hz, 2 H), 7.73 (d, J = 8.8 Hz, 2 H), 10.14 (bs, 1 H), 10.55 (bs, 1 H). ^13^C-NMR (DMSO-d6), δ: 170.2, 143.4, 140.7, 140.5, 138.5, 136.6, 133.9, 129.5, 129.4, 128.6, 128.3, 128.1, 127.9, 127.1, 125.5, 124.3, 120.5, 119.8, 119.2, 43.7. UPLC-MS (Method C): R_t_ 1.96 min, MS [ESI, m/z]: 443.2 [M + H]. Anal. calcd for C_26_H_22_N_2_O_3_S: C, 70.57; H, 5.01; N, 6.33. Found: C, 70.40; H, 5.27; N, 6.17.

### General method for the preparation of *N*-((4-(4-arylpiperazin-1-yl)phenyl)carbamothioyl) arylamides 48–58 and *N*-((4-(4-(arylsulfonyl)piperazin-1-yl)phenyl)carbamothioyl)arylamides 59–60

The appropriate aryl chloride **68a-72a** (1 mmol) and ammonium thiocyanate (1 mmol) were dissolved in acetone (4 mL/mmol) at 0 °C. Stirring was continued for 1 h at r.t., then the formed precipitate (NH_4_Cl) was filtered off. To the freshly filtered solution, the appropriate aromatic amine **62b–67b** (1 mmol) was added. The mixture was then stirred under reflux for 1 h. Upon completion of reaction, the resulting precipitate was collected by filtration. The crude product was purified by trituration, re-crystallisation or flash column chromatography to afford the title compound.

### 3-Chloro-*N*-((4-(4-(thiophene-2-carbonyl)piperazin-1-yl)phenyl)carbamothioyl)benzo[b]thiophene-2-carboxamide (48)

Purified by trituration from acetone. Obtained in 58% yield as a yellow solid. ^1^H-NMR (DMSO-d6), δ: 11.95 (bs, 1 H), 11.29 (bs, 1 H), 8.19 (dd, J_1_=7.9 Hz, J_2_ = 2.0 Hz, 1 H), 7,98 (dd, J_1_ = 7.9 Hz, J_2_ = 2.3 Hz, 1 H) 7.79 (dd, J_1_ = 5.0 Hz, J_2_ = 1.2 Hz, 1 H), 7.70–7.63 (m, 2 H), 7.57 (d, J = 9.0 Hz, 2 H), 7.48 (dd, J_1_ = 3.7 Hz, J_2_ = 1.1 Hz, 1 H), 7.16 (dd, J_1_ = 5.5 Hz, J_2_ = 3.6 Hz, 1 H), 7.02 (d, J = 9.0 Hz, 2 H), 3.81–3.79 (m, 4 H), 3.28–3.26 (m, 4 H). ^13^C-NMR (DMSO-d6), δ: 177.4, 162.8, 161.2, 149.4, 138.0, 137.5, 136.2, 130.2, 130.1, 129.9, 129.7, 128.8, 127.6, 126.8, 125.6, 124.1, 123.5, 122.5, 115.8, 48.7. UPLC-MS (Method C): R_t_ 2.18 min, MS [ESI, m/z]: 541.0, 543.0 [M + H]. Anal. Calcd for C_25_H_21_ClN_4_O_2_S_3_: C, 55.49; H, 3.91; N, 10.35. Found: C, 55.70; H, 4.08; N, 10.22^39^.

### *N*-((4-(4-(Thiophene-2-carbonyl)piperazin-1- yl)phenyl)carbamothioyl)benzo[b]thiophene-2-carboxamide (49)

Re-crystallised from MeOH/H_2_O. Obtained in 45% yield as a yellow solid. ^1^H-NMR (DMSO-d6), δ: 12.25 (bs, 1 H), 11.76 (bs, 1 H), 8.76 (s, 1 H), 8.10 (dd, J_1_=8.7 Hz, J_2_=0.8 Hz, 1 H), 8.02 (d, J = 7.8 Hz, 1 H), 7.79 (dd, J_1_ = 5.0 Hz, J_2_ = 1.1 Hz, 1 H), 7.56–7.48 (m, 3 H), 7.52–7.50 (m, 1 H), 7.49–7.48 (m, 1 H), 7.16 (dd, J_1_ = 5.2 Hz, J_2_ = 3.6 Hz, 1 H), 7.00 (d, J = 9.1 Hz, 2 H), 3.81–3.79 (m, 4 H), 3.27–3.25 (m, 4 H). ^13^C-NMR (DMSO-d6), δ: 178.4, 176.6, 163.0, 162.8, 149.4, 141.9, 139.4, 137.5, 137.1, 130.3, 130.1, 129.7, 127.9, 127.6, 126.7, 125.8, 125.7, 123.4, 115.9, 48.8. UPLC-MS (Method C): R_t_ 2.02 min, MS [ESI, m/z]: 507.1[M + H]. Anal. Calcd for C_25_H_22_N_4_O_2_S_3_: C, 59.27; H, 4.38; N, 11.06. Found: C, 59.34; H, 4.23; N, 10.92.

### *N*-((4-(4-(Thiophene-2-carbonyl)piperazin-1-yl)phenyl)carbamothioyl)-3- (trifluoromethyl)benzo[b] thiophene-2-carboxamide (50)

The crude product was purified by automated flash column chromatography (Biotage Isolera One, SNAP KP Sil 10 g) eluting with *n*-hexane:DCM:MeOH 100:0:0 v/v increasing to 0:90:10 v/v in 20 CV. Obtained in 41% yield as a yellow solid. ^1^H-NMR (DMSO-d6), δ: 12.40 (bs, 1 H), 11.94 (bs, 1 H), 8.27–8.24 (m, 1 H), 7.99–7.96 (m, 1 H), 7.79 (dd, J_1_ = 6.1 Hz, J_2_=1.1 Hz, 1 H), 7.73–7.70 (m, 2 H), 7.66–7.63 (m, 2 H), 7.56 (d, J=9 Hz, 1 H), 7.16 (dd, J_1_ = 7.1 Hz, J_2_=3.6 Hz, 1 H), 7.01 (d, J = 9.1 Hz, 2 H), 3.82–3.80 (m, 4 H), 3.28–3.26 (m, 4 H). ^13^C-NMR (DMSO-d6), δ: 177.6, 176.6, 172.6, 163.1, 162.8, 162.6, 149.4, 138.8, 137.5, 133.9, 130.1, 129.8, 129.7, 127.6, 126.9, 125.7, 124.0, 123.3, 115.8, 100.0, 55.4, 48.7. ^19^F-NMR (DMSO-d6), δ ppm: −56.59 (s, 3 F). UPLC-MS (Method C): R_t_ 2.05 min, MS [ESI, m/z]: 575.1 [M + H]. Anal. Calcd for C_26_H_21_F_3_N_4_O_2_S_3_: C, 54.34; H, 3.68; N, 9.75. Found: C, 54.11; H, 3.53; N, 9.98.

### N-((4-(4-(Thiophene-2-carbonyl)piperazin-1- yl)phenyl)carbamothioyl)benzamide (51)

The desired product did not precipitate after cooling the reaction at r.t., as the precipitated solid was in this case by-product **73**, which was collected by filtration and triturated from acetone. The filtrate was dried under vacuum and the crude product was purified by automated flash column chromatography (Biotage Isolera One, SNAP KP Sil 10 g) eluting with *n*-hexane:DCM:MeOH 100:0:0 v/v increasing to 0:95:5 v/v in 20 CV. Obtained in 42% yield as a yellow solid. ^1^H-NMR (CDCl_3_), δ: 12.47 (bs, 1 H), 9.09 (bs, 1 H), 7.93–7.91(m, 2 H), 7.70–7.67 (m, 1 H), 7.64–7.62 (m, 2 H), 7.60–7.56 (m, 2 H), 7.50 (dd, J_1_ = 5.0 Hz, J_2_ = 1.1, 1 H),7.38 (dd, J_1_=4.8 Hz, J_2_ = 1.1, 1 H), 7.10 (dd, J_1_ = 5.0 Hz, J_2_ = 3.6, 1 H), 7.00–6.97 (m, 2 H), 3.95 (t, 4 H), 3.30 (t, 4 H). ^13^C-NMR (CDCl_3_), δ: 178.2, 176.6, 166.9, 163.7, 149.5, 133.7, 131.7, 130.2, 129.2, 129.0, 128.8, 127.5, 126.8, 125.3, 116.3, 49.4. UPLC-MS (Method B): R_t_ 2.18 min, MS [ESI, m/z]: 451.1 [M + H]. Anal. Calcd for C_23_H_22_N_2_O_4_S_2_: C, 61.31; H, 4.92; N, 12.43. Found: C, 61.20; H, 5.13; N, 12.29.

Isolated by-product *N*-(4-(4-(thiophene-2-carbonyl)piperazin-1-yl)phenyl)benzamide (**73**)

Obtained in 37% yield as a yellow solid. ^1^H-NMR (CDCl_3_), δ: 10.35 (bs, 1 H), 8.03–8.02 (m, 2 H), 7.88–7.86 (m, 3 H), 7.66–7.57 (m, 5 H), 7.57 (bs, 1 H), 7.22 (dd, J_1_=1.2 Hz, J_2_=3.7 Hz, 1 H), 4.06 (m, 4 H), 3.49 (m, 4 H).^13^C-NMR (CDCl_3_), δ: 176.6, 165.8, 162.9, 137.1, 135.2, 132.1, 130.4, 129.9, 128.8, 128.1, 127.6, 121.8, 37.7. UPLC-MS (Method B): R_t_ 1.92 min, MS [ESI, m/z]: 392.3 [M + H].

### 4-Methyl-N-((4-(4-(thiophene-2-carbonyl)piperazin-1- yl)phenyl)carbamothioyl)benzamide (52)

The crude product was purified by trituration from acetone. Obtained in 78% yield as a yellow solid. ^1^H-NMR (DMSO-d6), δ: 12.54 (bs, 1 H),11.37 (bs, 1 H), 7.90 (d, J=8.2 Hz, 2 H), 7.79 (dd, J_1_ = 6.4 Hz, J_2_ = 1.1 Hz, 1 H), 7.55 (d, J = 9.1 Hz, 2 H), 7.48 (dd, J_1_ = 4.2 Hz, J_2_ = 1.1 Hz, 1 H), 7.34 (d, J = 8.2 Hz, 2 H, H- aromatic), 7.16 (dd, J_1_ = 6.4 Hz, J_2_ = 4.2 Hz, 1 H, H-aromatic), 7.00 (d, J = 9.1 Hz, 2 H, H- aromatic), 3.81–3.80 (m, 4 H), 3.27–3.25 (m, 4 H), 2.40 (s, 3 H). ^13^C-NMR (DMSO-d6), δ: 179.1, 176.6, 168.5, 162.8, 149.3, 144.0, 139.7, 137.5, 130.1, 129.7, 129.5, 129.2, 127.6, 125.6, 115.9, 48.8, 21.6. UPLC-MS (Method B): R_t_ 1.97 min, MS [ESI, m/z]: 465.2 [M+H]. Anal. Calcd for C_24_H_24_N_4_O_2_S_2_: C, 62.05; H, 5.21; N, 12.06. Found: C, 62.27; H, 4.97; N, 11.99.

### 3-Chloro-*N*-((4-(4-(furan-2-carbonyl)piperazin-1-yl)phenyl)carbamothioyl)benzo[b]thiophene-2-carboxamide (53)

The crude product was purified by trituration from acetone. Obtained in 74% yield as a light orange solid. ^1^H-NMR (CDCl_3_), δ: 12.11 (bs, 1 H), 10.08 (bs, 1 H), 7.92–7.90 (m, 1 H), 7.83–7.82 (m, 1 H), 7.55–7.48 (m, 4 H), 7.45–7.44 (m, 1 H), 6.99 (dd, J_1_=4.4 Hz, J_2_ = 1.4 Hz, 1 H), 6.91–6.88 (m, 2 H), 6.44 (dd, J_1_=5.2 Hz, J_2_ = 1.4 Hz,), 3.91 (m, 4 H), 3.23–3.21 (m, 4 H). ^13^C-NMR (CDCl_3_), δ: 177.4, 176.6, 160.1, 159.1, 149.5, 147.9, 143.8, 138.9, 136.8, 129.9, 128.8, 126.1, 125.2, 123.9, 122.9, 122.4, 116.8, 116.3, 111.4, 49.3. UPLC-MS (Method C): R_t_ 2.14 min, MS [ESI, m/z]: 525.1 [M + H]. Anal. Calcd for C_25_H_21_ClN_4_O_3_S_2_: C, 57.19; H, 4.03; N, 10.67. Found: C, 56.93; H, 3.88; N, 10.91.

### *N*-((4-(4-Benzoylpiperazin-1-yl)phenyl)carbamothioyl)-3-chlorobenzo[b]thiophene-2-carboxamide (54)

The crude product was purified by trituration from acetone. Obtained in 53% yield as a yellow solid. ^1^H-NMR (CDCl_3_), δ: 12.20 (bs, 1 H), 10.17 (bs, 1 H), 8.02–8.00 (m, 1 H), 7.93–7.91 (m, 1 H), 7.64–7.57 (m, 4 H), 7.48–7.46 (m, 5 H), 7.00–6.96 (m, 2 H), 3.97 (m, 2 H), 3.64 (m, 2 H), 3.32–3.20 (m, 4 H). ^13^C-NMR (CDCl_3_), δ: 177.2, 170.4, 160.2, 149.6, 138.9, 136.8, 135.5, 130.1, 129.9, 129.6, 128.9, 128.6, 127.1, 126.1, 125.2, 124.0, 122.9, 122.5, 116.5, 49.1. UPLC-MS (Method C): R_t_ 2.18 min, MS [ESI, m/z]: 535.1 [M + H]. Anal. Calcd for C_27_H_23_ClN_4_O_2_S_2_: C, 60.61; H, 4.33; N, 10.47. Found: C, 60.38; H, 4.51; N, 10.29.

### *N*-((4-(4-Benzoylpiperazin-1-yl)phenyl)carbamothioyl)benzamide (55)

The crude product was purified by trituration from acetone. Obtained in 62% yield as a white solid. ^1^H-NMR (DMSO-d6), δ: 10.28 (bs, 1 H), 7.97–7.95 (m, 2 H), 7.77 (d, J = 8.6 Hz, 2 H), 7.61 (m, 1 H), 7.55–7.52 (m, 2 H), 7.49–7.47 (m, 6 H), 7.39 (bs, 2 H), 3.91 (m, 2 H), 3.65 (m, 2 H), 3.36 (m, 4 H). ^13^C-NMR (DMSO-d6), δ: 178.5, 176.6, 169.5, 165.8, 135.9, 135.3, 132.0, 130.2, 128.9, 128.8, 128.1, 127.5, 121.8, 36.4. UPLC-MS (Method C): R_t_ 1.89 min, MS [ESI, m/z]: 445.2 [M + H]. Anal. Calcd for C_25_H_24_N_4_O_2_S: C, 67.55; H, 5.44; N, 12.60. Found: C, 67.61; H, 5.17; N, 12.47.

### 3-Chloro-*N*-((4-(4-(thiazole-2-carbonyl)piperazin-1-yl)phenyl)carbamothioyl)benzo[b]thiophene-2-carboxamide (56)

The crude product was purified by trituration from acetone. Obtained in 43% yield as an off-white solid. ^1^H-NMR (CDCl_3_), δ: 12.20 (bs, 1 H) 10.17 (bs, 1 H), 8.01–8.01 (m, 1 H),7.93 (d, J = 3.2 Hz, 1 H), 7.93–7.91 (m, 1 H), 7.65–7.57 (m, 5 H), 7.02–6.99 (m, 2 H), 4.64 (m, 2 H), 4.00 (m, 2 H), 3.36 (m, 4 H). ^13^C-NMR (CDCl_3_), δ: 177.2, 176.6, 165.0, 160.1, 154.2, 149.6, 143.2, 138.9, 136.8, 129.9, 129.6, 128.9, 126.1, 125.2, 124.2, 123.9, 122.9, 116.3, 49.6, 49.1, 46.1, 43.3. UPLC-MS (Method C): R_t_ 2.20 min, MS [ESI, m/z]: 543.0 [M + H]. Anal. Calcd for C_24_H_20_ClN_5_O_2_S_3_: C, 53.18; H, 3.72; N, 12.92. Found: C, 52.96; H, 3.79; N, 13.03.

### N-((4-(4-Benzoylpiperazin-1-yl)phenyl)carbamothioyl)-4-methylbenzamide (57)

The desired product did not precipitate after cooling the reaction at r.t., as the precipitated solid was in this case by-product **74**, which was collected by filtration and triturated from acetone. The filtrate was dried under vacuum and the crude product was purified by automated flash column chromatography (Biotage Isolera One, SNAP KP Sil 10 g) eluting with *n*-hexane:DCM:MeOH 100:0:0 v/v increasing to 0:95:5 v/v in 25 CV. Obtained in 41% yield as a yellow solid. ^1^H-NMR (CDCl_3_), δ: 12.50 (bs 1 H), 9.05 (bs 1 H), 7.80 (d, J = 8.3 Hz, 2 H), 7.61 (d, J = 8.9 Hz, 2 H), 7.35 (d, J = 7.9 Hz, 2 H), 7.48–7.46 (m, 5 H), 6.99–6.95 (m, 2 H), 3.97 (m, 2 H), 3.63 (m, 2 H), 3.31–3.19 (m, 4 H), 2.38 (s, 3 H). ^13^C-NMR (CDCl_3_), δ: 178.3, 170.4, 166.8, 149.5, 144.7, 135.6, 130.4, 129.9, 129.8, 128.9, 128.5, 127.5, 127.1, 125.2, 116.5, 49.5, 21.6. UPLC-MS (Method C): R_t_ 2.29 min, MS [ESI, m/z]: 459.2 [M + H]. Anal. Calcd for C_26_H_26_N_4_O_2_S: C, 68.10; H, 5.72; N, 12.22. Found: C, 68.25; H, 5.53; N, 11.97.

Isolated by-product *N*-(4-(4-benzoylpiperazin-1-yl)phenyl)-4-methylbenzamide (**74**)

Obtained in 35% yield as a yellow solid. ^1^H-NMR (CDCl_3_), δ: 10.20 (bs, 1 H), 7.88 (d, J = 8.2 Hz, 2 H), 7.78 (d, J = 8.5 Hz, 2 H), 7.50–7.47 (m, 6 H), 7.37–7.33 (m, 3 H), 3.92 (m, 2 H), 3.63 (m, 2 H), 3.36 (m, 4 H), 2.39 (s, 3 H). ^13^C-NMR (CDCl_3_), δ: 195.6, 176.6, 169.5, 165.6, 142.0, 135.9, 132.4, 130.2, 129.4, 129.3, 128.1, 127.6, 121.8, 38.1, 21.5. (Method C): R_t_ 1.75 min, MS [ESI, m/z]: 400.3 [M + H].

### *N*-((4-(4-(Thiazole-2-carbonyl)piperazin-1- yl)phenyl)carbamothioyl)benzamide (58)

The desired product did not precipitate after cooling the reaction at r.t., as the precipitated solid was in this case by-product **75**, which was collected by filtration and triturated from acetone. The filtrate was dried under vacuum and the crude product was purified by automated flash column chromatography (Biotage Isolera One, SNAP KP Sil 10 g) eluting with *n*-hexane:DCM:MeOH 100:0:0 v/v increasing to 0:90:10 v/v in 20 CV. Obtained in 47% yield as a yellow solid. ^1^H-NMR (DMSO-d_6_), δ: 12.46 (bs, 1 H), 9.07 (bs, 1 H), 7.94–7.91 (m, 3 H), 7.70–7.67 (m, 1 H), 7.65–7.62 (m, 2 H), 7.59–7.56 (m, 3 H), 7.02–7.09 (m, 2 H), 4.64 (m, 2 H), 4.01 (m, 2 H), 3.55 (m, 4 H). ^13^C-NMR (DMSO-d_6_), δ: 178.1, 166.9, 165.1, 159.2, 149.5, 149.6, 143.2, 133.7, 131.8, 129.2, 127.5, 125.3, 124.1, 116.3, 49.7. UPLC-MS (Method C): R_t_ 1.89 min, MS [ESI, m/z]: 452.2 [M + H]. Anal. Calcd for C_22_H_21_N_5_O_2_S_2_: C, 58.52; H, 4.69; N, 15.51. Found: C, 58.48; H, 4.87; N, 15.34.

Isolated by-product *N*-(4-(4-(thiazole-2-carbonyl)piperazin-1-yl)phenyl)benzamide (**75**)

Obtained in 29% yield as a yellow solid. ^1^H-NMR (DMSO-d_6_), δ: 10.29 (bs, 1 H), 8.07 (m, 2 H), 7.98–7.96 (m, 2 H), 7.77 (d, J = 8.9 Hz, 2 H), 7.61–7.58 (m, 1 H), 7.55–7.51 (m, 2 H), 7.37–7.34 (m, 2 H), 4.71–4.59 (m, 4 H), 3.99 (m, 2 H), 3.43 (m, 2 H). ^13^C-NMR (DMSO-d_6_), δ: 176.7, 172.4, 165.7, 164.8, 158.9, 144.0, 135.4, 131.9, 128.8, 128.1, 126.1, 121.9, 31.1. (Method B): R_t_ 1.89 min, MS [ESI, m/z]: 393.2 [M + H].

### *N*-((4-(4-(Thiophen-2-ylsulfonyl)piperazin-1- yl)phenyl)carbamothioyl)benzamide (59)

The desired product did not precipitate after cooling the reaction at r.t., therefore the reaction mixture was dried under vacuum and the crude product was purified by automated flash column chromatography (Biotage Isolera One, SNAP KP Sil 10 g) eluting with *n*-hexane:DCM:MeOH 100:0:0 v/v increasing to 0:95:15 v/v in 20 CV. Obtained in 69% yield as a yellow solid. ^1^H-NMR (DMSO-d_6_), δ: 12.47 (bs, 1 H), 11.47 (bs, 1 H), 8.09 (dd, J_1_ = 6.3 Hz, J_2_ = 1.3, 1 H), 7.98–7.96 (m, 2 H), 7.00 (dd, J_1_ = 3.8 Hz, J_2_ = 1.3, 1 H), 7.68–7.64 (m, 1 H), 7.56–7.51 (m, 4 H), 7.32 (dd, J_1_=6.3 Hz, J_2_ = 3.8, 1 H), 6.96 (d, J = 9.1 Hz, 2 H), 3.30–3.28 (m, 4 H), 3.09–3.07 (m, 4 H). ^13^C-NMR (DMSO-d_6_), δ: 179.1, 176.6, 168.7, 148.9, 134.8, 134.6, 133.8, 133.5, 132.7, 130.5, 129.1, 128.9, 125.6, 116.3, 48.2, 46.3. UPLC-MS (Method C): R_t_ 2.00 min, MS [ESI, m/z]: 487.1 [M + H]. Anal. Calcd for C_22_H_22_N_4_O_3_S_3_: C, 54.30; H, 4.56; N, 11.51. Found: C, 54.45; H, 4.36; N, 11.44.

### *N*-((4-(4-(Phenylsulfonyl)piperazin-1-yl)phenyl)carbamothioyl)benzamide (60)

The desired product did not precipitate after cooling the reaction at r.t., as the precipitated solid was in this case by-product **76**, which was collected by filtration and triturated from acetone. The filtrate was dried under vacuum and the crude product was purified by automated flash column chromatography (Biotage Isolera One, SNAP KP Sil 25 g) eluting with *n*-hexane:DCM:MeOH 100:0:0 v/v increasing to 0:95:5 v/v in 20 CV. Obtained in 77% yield as a yellow solid. ^1^H-NMR (DMSO-d_6_), δ: 12.45 (bs, 1 H), 11.46 (bs, 1 H), 7.98–7.96 (m, 2 H), 7.81–7.75 (m, 3 H), 7.71–7.64 (m, 3 H), 7.55–7.50 (m, 4 H), 6.94 (d, J=9.1 Hz, 2 H), 3.26–3.24 (m, 4 H), 3.04–3.02 (m, 4 H). ^13^C-NMR (DMSO-d_6_), δ: 207.4, 199.5, 180.8, 179.0, 176.6, 138.1, 135.1, 133.9, 130.0, 129.1, 128.9, 128.1, 125.6, 116.3, 48.3, 46.2. UPLC-MS (Method B): R_t_ 2.37 min, MS [ESI, m/z]: 481.2 [M + H]. Anal. Calcd for C_24_H_24_N_4_O_3_S_2_: C, 59.98; H, 5.03; N, 11.66. Found: C, 59.76; H, 4.91; N, 11.90.

Isolated by-product *N*-(4-(4-benzoylpiperazin-1-yl)phenyl)benzamide (**76**)

Obtained in 14% yield as a yellow solid. ^1^H-NMR (DMSO-d_6_), δ: 10.19 (bs, 1 H), 7.95–7.93 (m, 2 H), 7.81–7.76 (m, 3 H), 7.71–7.64 (m, 4 H), 7.59–7.56 (m, 1 H), 7.53–7.50 (m, 2 H), 7.22–7.00 (m, 2 H), 3.39–3.24 (m, 4 H), 3.17–3.07 (m, 4 H).^13^C-NMR (DMSO-d_6_), δ: 176.6, 165.6, 162.6, 135.4, 135.0, 134.0, 131.9, 130.0, 128.8, 128.1, 128.0, 121.8, 118.0, 49.8, 46.0. (Method B): R_t_ 2.15 min, MS [ESI, m/z]: 422.2 [M + H].

## Biology

### RdRp enzyme inhibition assays

#### Quantitative RdRp activity and gel-based assays

Fluorescent RdRp activity assays were carried out as previously described^12,29^. Briefly, RdRp activity was quantified by monitoring the formation of double-stranded RNA (dsRNA) from a single stranded homopolymeric template, poly(C) (Sigma Aldrich), using the fluorescent dye PicoGreen (Life Technologies, Carlsbad, CA, USA). RdRp assays were performed in 384-well plates, and each reaction mixture contained 400 ng enzyme, 45 μM GTP, 10 ng/μL poly(C) RNA, 2.5 mM MnCl2, 5 mM dithiothreitol, (DTT), 0.01% bovine serum albumin (BSA), and 0.005% Tween 20 in 20 mM Tris-HCl, pH 7.5, with a final reaction volume of 25 μL. The PPNDS and compound 1 were used as a positive controls. RdRps were incubated for 10 mins at 30 °C in the presence of the test compounds or the compound vehicle DMSO (0.5% vol/vol) before addition into the reaction mixture, which was then allowed to run 15 min at 30 °C then terminated with 10 mM EDTA, followed by PicoGreen staining and dsRNA quantitation. GraphPad Prism V6.05 (La Jolla, CA, USA) was used to plot the IC_50_ values. A secondary gel-based polymerase activity assay was used as a counter-screen as described below, to exclude the possibility of RdRp activity enhancement. Primed elongation activity was examined in a gel-based assay as previously described^12,29^ using the RNA template (PE44-NoV). Gels were imaged using BioRad (Hercules, CA, U.SA) Geldoc Universal Hood II, running BioRad Image Lab software, V4.1, build 16.

### MNV and HuNoV cell-based assays

#### Norovirus

Cells and virus: MNV (virus strain MNV-1.CW1) was propagated in RAW 264.7 cells grown in DMEM (Life Technologies, Gent, Belgium) supplemented with 10% or 2% FBS, 2 mM L-glutamine, 20 mM HEPES, 0.075 g/L sodium bicarbonate, 1 mM sodium pyruvate, 100 U penicillin/mL and 100 lg/mL streptomycin at 37 °C in a humidified atmosphere of 5% CO_2_. The human norovirus GI.1 replicon harboring gastric tumor-1 cell line (HGT-NV) (kindly provided by Dr. Ian Goodfellow, University of Cambridge) was maintained in DMEM supplemented with 10% FBS, 2mM L-glutamine, 0.075 g/L sodium bicarbonate and 1.0 mg/ml of Geneticin (G418; Life Technologies), at 37 °C in a humidified atmosphere of 5% CO_2_^34^.

Antiviral assay with MNV: The antiviral activity of the Compounds was determined using an MTS [3-(4,5-dimethylthiazol-2-yl)-5-(3-carboxymethoxyphenyl)-2-(4-sulfophenyl)-2H-tetrazolium]-based cytopathic effect (CPE) reduction assay. RAW 264.7 cells (1 × 10^4^ cells/well) were seeded in a 96-well plate and infected with MNV (MOI of 0.001) in the presence (or absence) of a dilution series of Compounds Cells were incubated for 3 days, i.e. until complete CPE was observed in infected untreated cells. Then, a MTS-phenazinemethosulfate (MTS/PMS) stock solution [(2 mg/mL MTS (Promega, Leiden, The Netherlands) and 46 g/mL PMS (Sigma–Aldrich, Bornem, Belgium) in PBS at pH 6–6.5)] was diluted 1/20 in MEM (Life Technologies, Gent, Belgium) and 75 µL were added to each well. After 2 h, the optical density (OD) was read at 498 nm. The %CPE reduction was calculated as [(OD_treated_)_MNV_ − OD_VC_]/[OD_CC_ − OD_VC_] × 100, where OD_CC_ represents the OD of the uninfected untreated cells, whereas OD_VC_ and (OD_treated_)_MNV_ represent the OD of infected untreated cells and virus-infected cells treated with a compound concentration, respectively. The 50% effective concentration (EC_50_) was defined as the compound concentration that protected 50% of the cells from virus-induced CPE^34^.

Cytotoxicity: The cytotoxicity of the Compounds was evaluated by the MTS method, by exposing uninfected cells to the same concentrations of compounds for 3 days. The %cell viability was calculated as (OD_treated_/OD_CC_) × 100, where OD_CC_ is the OD of uninfected untreated cells and OD_treated_ are uninfected cells treated with compound. The CC_50_ was defined as the compound concentration that reduces the number of viable cells by 50%^34^.

Antiviral assay with the HGT-NV replicon: The inhibitory effect of Compounds on human norovirus replication was assessed by quantification of the levels of HuNoV GI replicon RNA and of the reference (housekeeping) gene β-actin mRNA [by quantitative reverse transcription polymerase chain reaction (qRT-PCR)]. To this end, HGT cells (2000 cells/well) were seeded in 96-well plates, in complete DMEM without the selection marker G418. Following an incubation period of 24 h, a serial dilution of rupintrivir was added to the cultures. After 72 h of incubation, cell monolayers were washed with phosphate-buffered saline (PBS) and collected for quantification of RNA load by qRT-PCR. Intracellular RNA was extracted from cells using the cell-to-cDNA lysis buffer (Ambion, Life Technologies). For detection of HuNoV GI replicon RNA, forward (5′-CCG GCT ACC TGC CCA TTC-3′), reverse (5′-CCA GAT CAT CCT GAT CGA CAA G-3′) primers and probe (5′-FAM-ACA TCG CAT CGA GCG AGC ACG TAC-TAMRA-3′) for the neomycin gene were used. For detection of β-actin mRNA, forward (5′-GGC ATC CAC GAA ACT ACC TT-3′), reverse (5′-AGC ACT GTG TTG GCG TAC AG-3′) primers and probe (5′-HEX-ATC ATG AAG TGT GAC GTG GAC ATC CG-BHQ1–3′) were used. One-step qRT-PCR was performed in a 20 µL reaction mixture containing 10 µl 2X iTaq Universal SYBR® Green one step reaction mix (Bio-rad, California, USA), 5.17 µl RNAse free water, 0.5 µl of iScript reverse transcriptase (Bio-rad, California, USA), 4 µl of the template RNA and either 300 nM of HuNoV GI replicon primers and probe, 300 nM of β-actin primers and 200 nM of probe. Cycling conditions were: reverse transcription at 50 °C for 10 min, initial denaturation at 95 °C for 3 min, followed by 40 cycles of denaturation at 95 °C for 15 s, annealing and extension at 60 °C for 30 seconds (Roche Lightcycler®96, Roche Diagnostics, Belgium). To determine the relative expression levels of HuNoV GI replicon RNA, β-actin was used as a normalizer and ratios were calculated using the Pfaffl method. The Expression Ratio (HuNoV GI Replicon/β-actin) was calculated as: Expression Ratio = (E _HuNoV_)^ΔCT, HuNoV (CC – TC)^/(E_β-actin_)^ΔCT, β-actin (CC – TC)^, where E _HuNoV_ and E_β-actin_ represent the amplification efficiency (E  = 10^–1/slope^) for the HuNoV GI replicon and β-actin qRT-PCR reactions, respectively. ΔCT, HuNoV (CC – TC) is the Ct of untreated control cells (CC) minus the Ct of cells treated with a compound concentration (TC) obtained with HuNoV GI replicon primers and probe. ΔCT, β-actin (CC – TC) is the Ct of untreated control cells (CC) minus the Ct of cells treated with a compound concentration (TC) obtained with β-actin primers and probe. Efficiency values (E _HuNoV_ and E_β-actin_) were determined for each qRT-PCR reaction. The 50% effective concentration (EC_50_) was defined as the compound concentration that resulted in a 50% reduction of the relative HuNoV replicon RNA levels^34^.

### Molecular modelling

All molecular docking studies were performed on a Viglen Genie Intel®Core^TM^ i7–3770 vPro CPU@ 3.40 GHz × 8 running Ubuntu 16.04. Molecular Operating Environment (MOE) 2018.10^33^ and Maestro (Schrödinger Release 2017–1)^33^ were used as molecular modelling software. The RdRp structure n complex with PPNDS was downloaded from the PDB data bank (http://www.rcsb.org/; PDB code 4LQ3). The protein was pre-processed using the Schrödinger Protein Preparation Wizard by assigning bond orders, adding hydrogens and performing a restrained energy minimization of the added hydrogens using the OPLS_2005 force field. Ligand structures were built with MOE and then prepared using the Maestro LigPrep tool by energy minimising the structures (OPLS_2005 force filed), generating possible ionization states at pH 7 ± 2, generating tautomers and low-energy ring conformers. An 11 Å docking grid (inner-box 10 Å and outer-box 21 Å) was prepared using as centroid the co-crystallised PPNDS. Molecular docking studies were performed using Glide SP precision keeping the default parameters and setting 5 as number of output poses per input ligand to include in the solution. The output poses were saved as mol2 file. The docking results were visually inspected for their ability to bind the active site.

The Flexible Alignment was performed using MOE2018.10. The MOE flexible alignment tool generates different possible conformations for each molecule present in the input database (in-house small-molecule database of reported non-nucleoside inhibitors of viral polymerases, previously prepared in MOE) that could overlap the conformation of the assigned template, which is kept rigid (compound 1). The quality of the alignment is evaluated by a score which is a sum of the internal strain of the obtained conformation (the smaller, the better) and the overlap of molecular features (aromatic regions, donors/acceptors). MOE, for each alignment performed, evaluates the average internal energy of the ligands U, the similarity score F (the lower value is, the better the two structures overlap) and the value S (sum of U and F values obtained for each alignment). A good alignment should present a dU value (the average strain energy of the molecules in the alignment in kcal/mol) lower than 1 kcal/mol meaning that the obtained conformations are not energetically disadvantaged. The obtained data from the Flexible Alignment with dU of 0.0 (no energy penalty) were kept and ranked according to the lowest S value. TPB (**48**) resulted the best match for its structural overlapping.

The conformation of **1** required for the Flexible Alignment was obtained using MOE conformational search tool, using the default parameters, selecting Stochastic as search method and setting RMS Gradient to 0.001 and the RMSD Limit to 0.15. 159 conformations were generated and among them the best in terms of dE (the strain of the conformation relative to the lowest energy conformation with the same stereochemistry configuration. Value of 0 indicate the best result) and E (value of the potential energy of the conformation) was chosen as template for the Flexible Alignment.

The Glide-Based Core Hopping tool of Maestro^32^ was used to perform a scaffold replacement. The structure of compound 1 docked into the RdRp binding site was used as template ligand keeping as fixed the two terminal hydrophobic rings and let the program replacing the original scaffold with new optimal linkers. The new ligands are then automatically docked on the receptor and ranked and scored according to the docking results. The default parameters were used, using as docking grid the one previously generated for the SP docking studies (see above).

## Supplementary information

Supplementary information
