# Supplementary material for: A new antiviral scaffold for human norovirus identified with computer-aided approaches on the viral polymerase
Source: Sci Rep. 2019 Dec 5;9:18413. doi: 10.1038/s41598-019-54903-7 (PMC6895199; doi:10.1038/s41598-019-54903-7)
Supplement: Supplementary file 1 — Supplementary information [file 41598_2019_54903_MOESM1_ESM.docx]

*Supplementary Information*

**A new antiviral scaffold for human norovirus identified with computer-aided approaches on the viral polymerase**

Gilda Giancotti^a^, Ilaria Rigo^a^, Gaia Pasqualetto^a^, Mark T. Young^b^, Johan Neyts^c^, Joana Rocha-Pereira^c^, Andrea Brancale^a^, Salvatore Ferla^a*^, Marcella Bassetto^a,d^

^a^Cardiff School of Pharmacy and Pharmaceutical Sciences, Cardiff, King Edward VII Avenue, Cardiff, CF103NB, UK

^b^Cardiff School of Biosciences, Sir Martin Evans Building, Museum Avenue, Cardiff, CF10 3AX, UK

^c^KU Leuven – Department of Microbiology, Immunology and Transplantation, Rega Institute, Laboratory of Virology and Chemotherapy, Leuven, Belgium.

^d^Department of Chemistry, Swansea University, Swansea, UK

* Correspondence and requests for material s should be addressed to Dr. S. Ferla (email: ferlas1@cardiff.ac.uk).

*Contents*

**Page S2** Figure S1

**Page S3** Figure S2

**Page S3-4** Figure S3

**Page S5** Figure S4

**Page S6** Table S1

**Page S7** Preparation and characterisation of synthetic intermediates

**Page S13** References


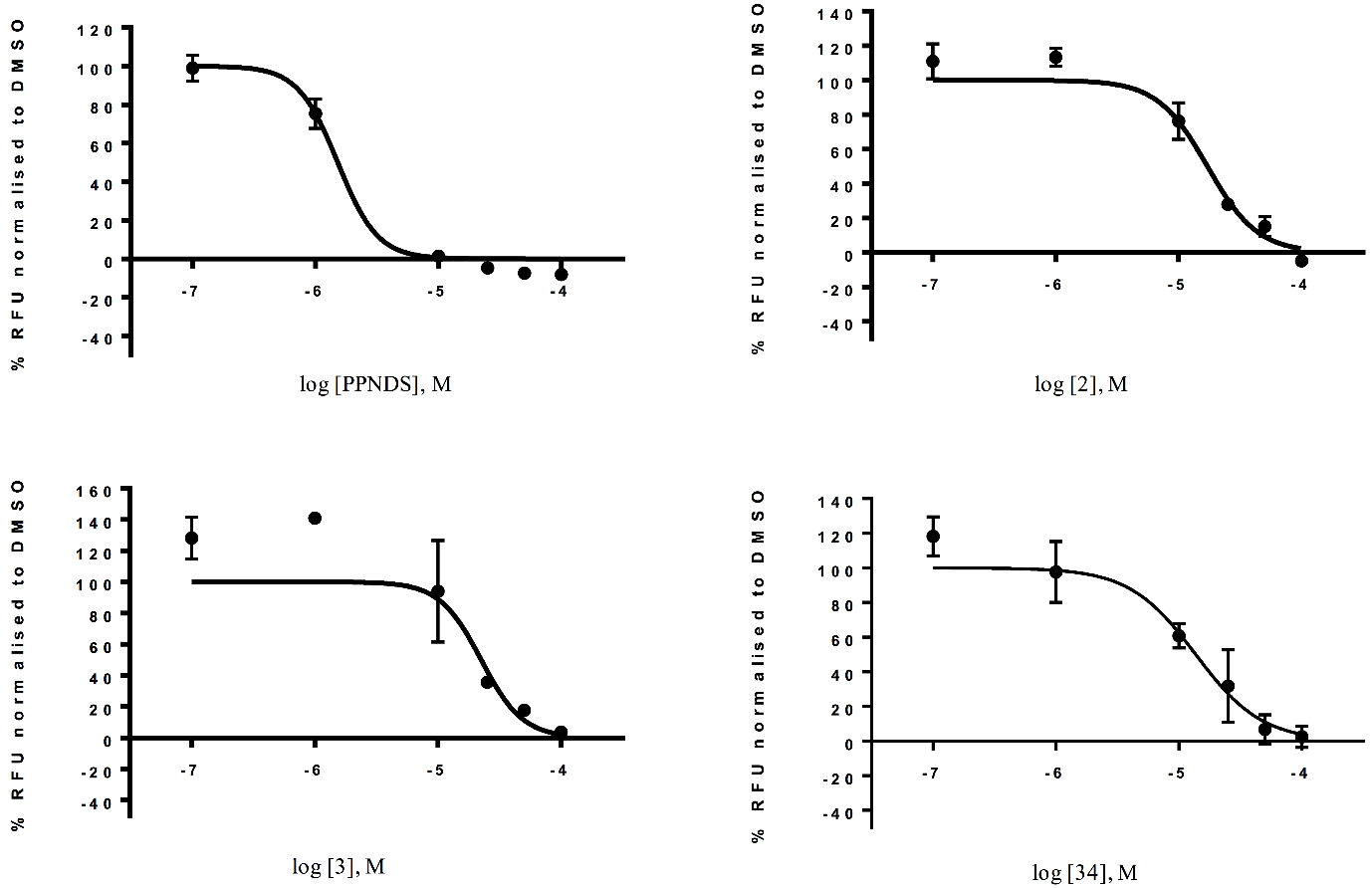


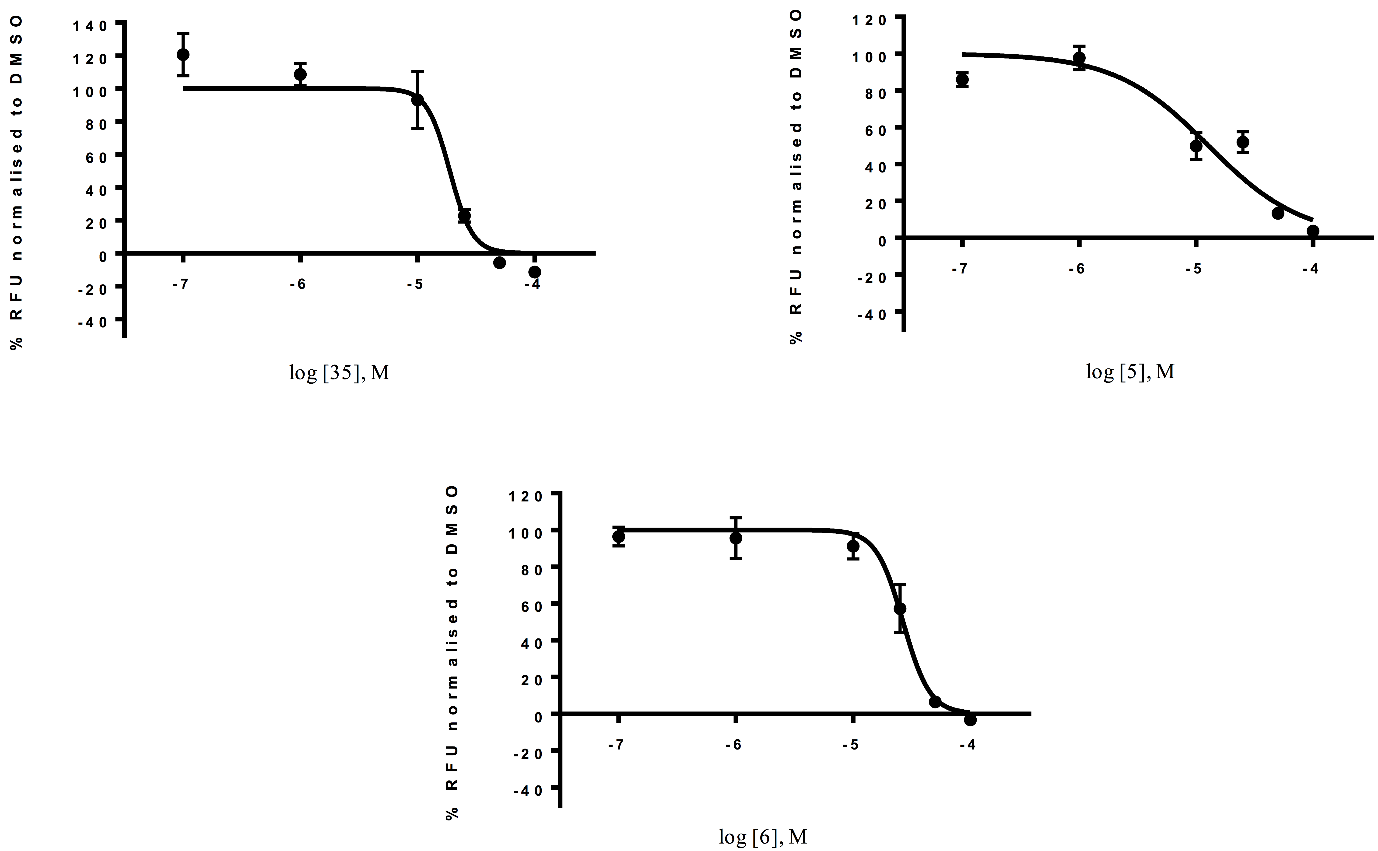


**Supplementary Figure S1. Representative dose-response curves of compounds reported in Table 1.** Example of dose-response curves for the enzymatic inhibition assay of human norovirus Sydney 2012 RdRp activity *in vitro*. Compounds were tested at six different concentrations (concentration range examined: 0.1-100µM) and compared to the relative activity of mock treated samples containing the vehicle only (0.5% DMSO [vol/vol]). Percentage of inhibition was normalised to control DMSO. IC_50_ values were calculated using GraphPad Prism V7.03.


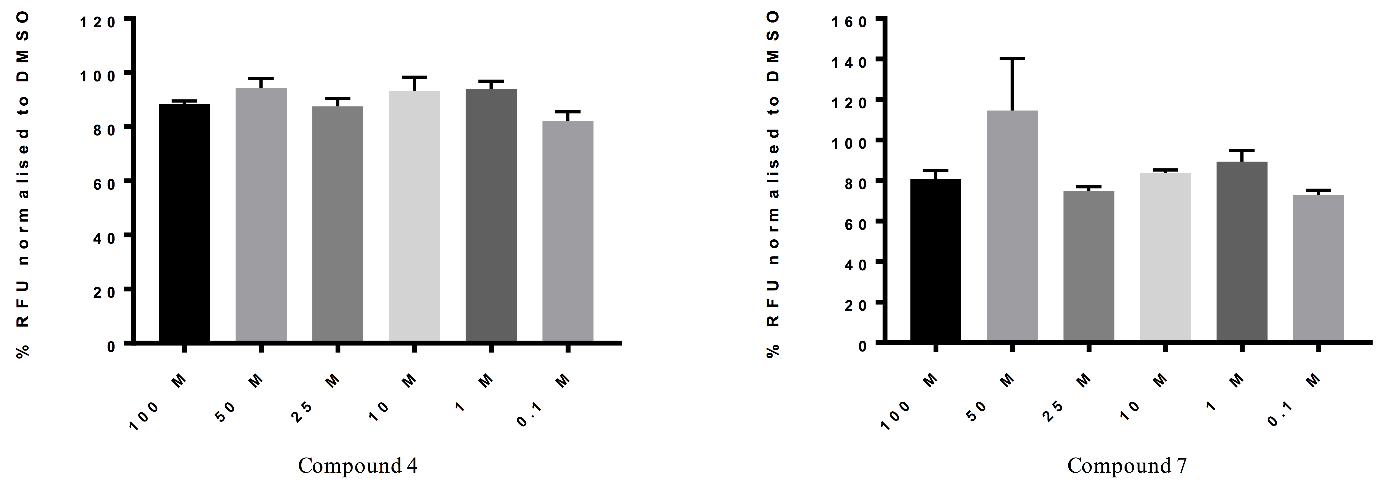


**Supplementary Figure S2. Percentage of inhibition of human norovirus Sydney 2012 RdRp activity *in vitro* at six different concentrations for compound 4 and 7.** Compound **4** reached only 15% inhibition at the maximum concentration tested, while 7 only reached 20% inhibition at the maximum concentration tested. For both compounds was not possible to attain an IC_50_ value. Percentage of inhibition was normalised to control DMSO.

**
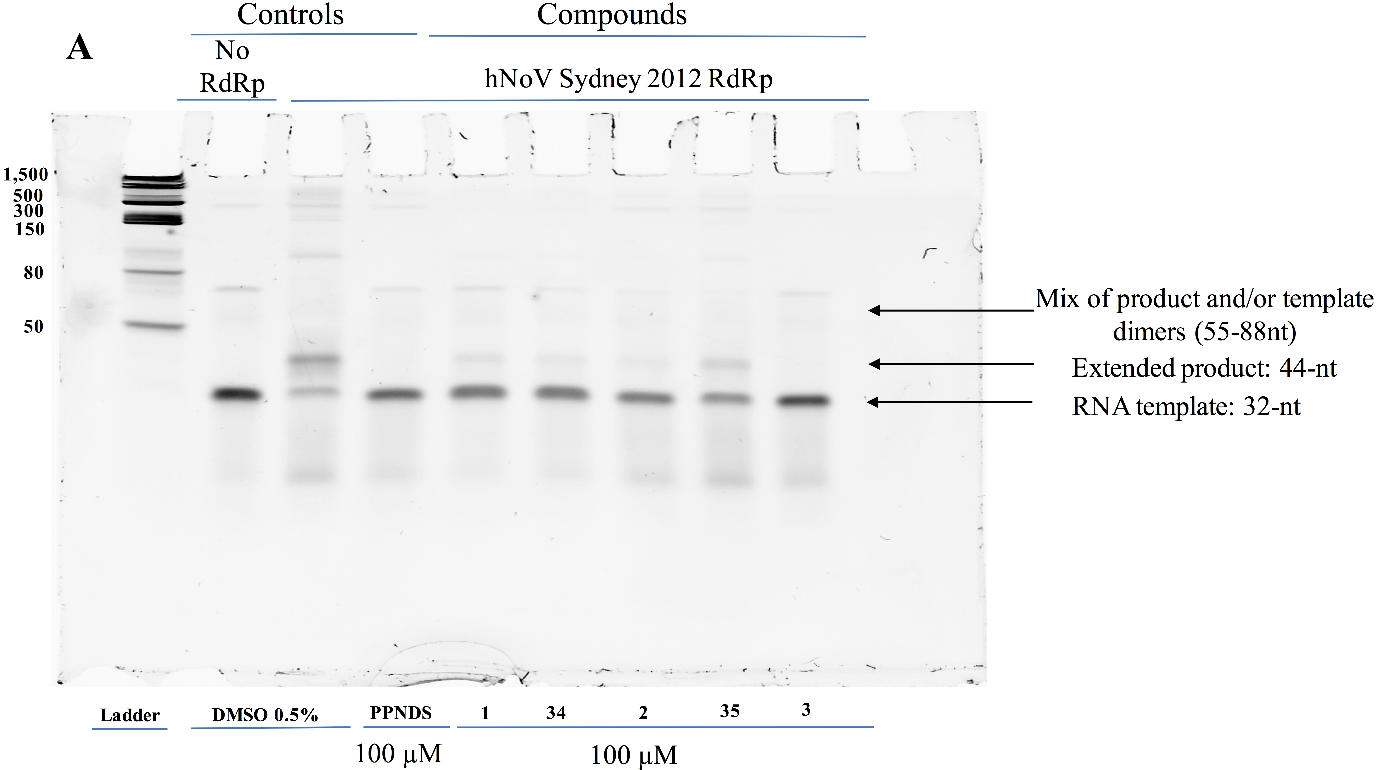
**

**
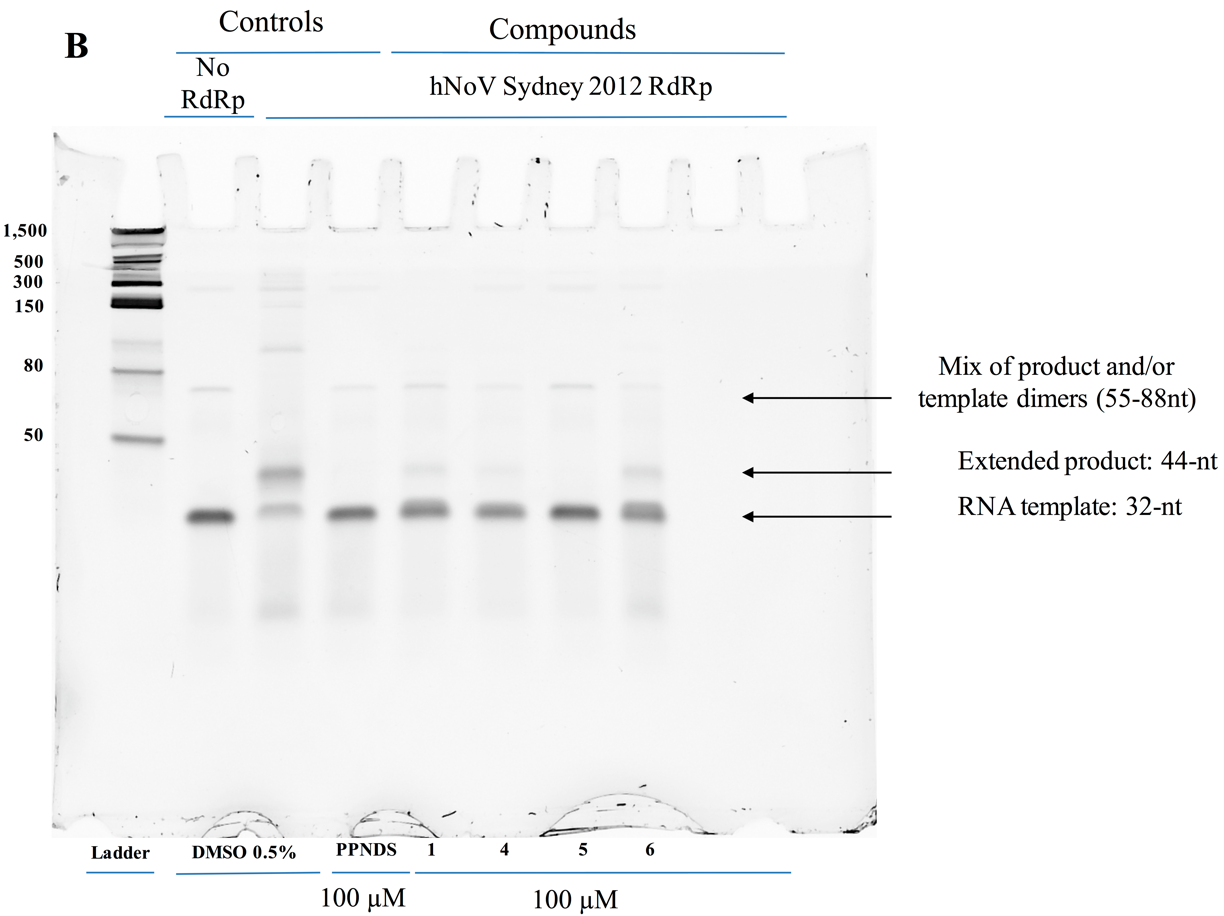
**

**
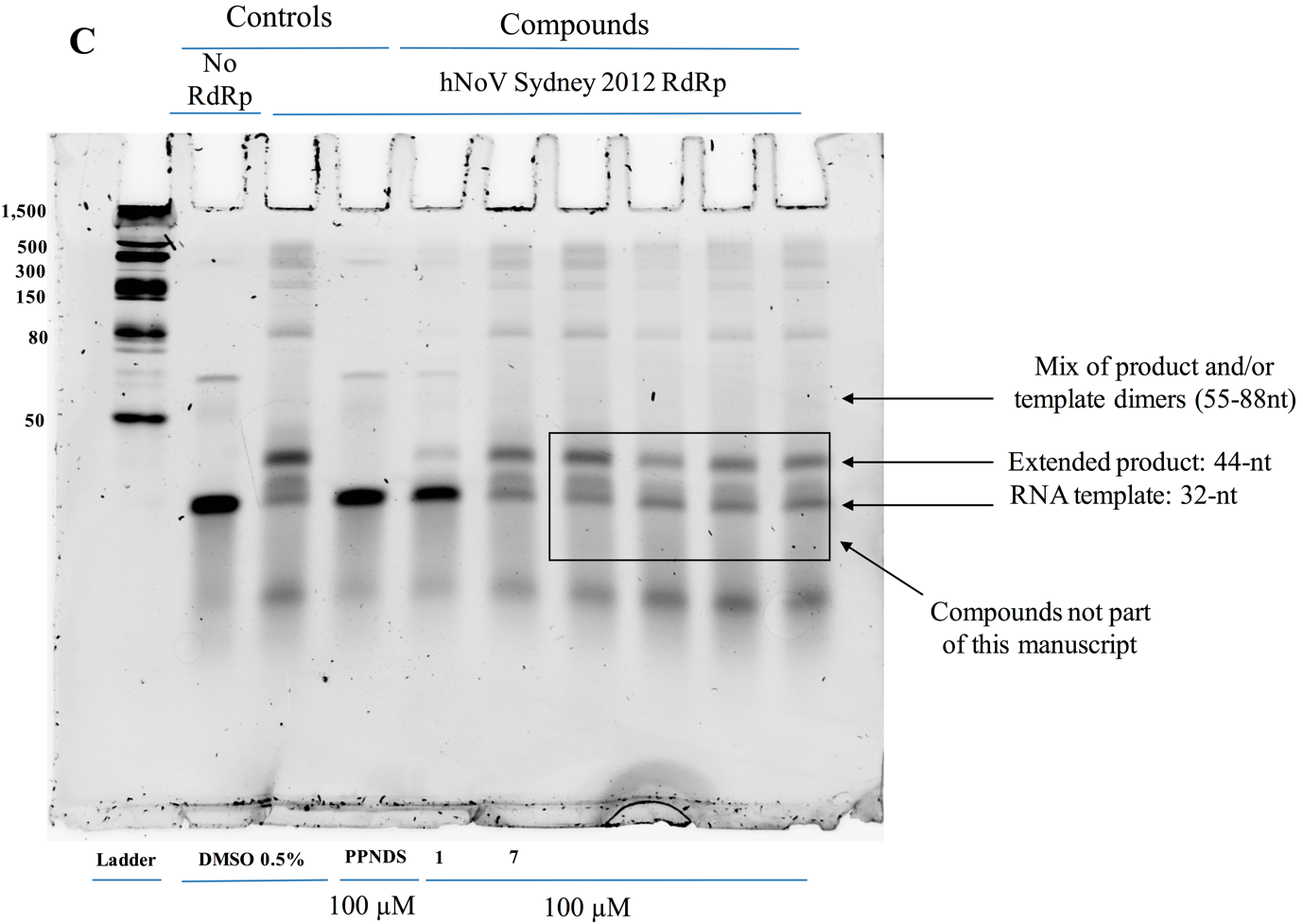
**

**Supplementary Figure S3 (A-C). Full length gel from Figure 3.** A counter-screen gel-shift assay was used to confirm norovirus RdRp inhibitory activity of the eight compounds. The eight compounds were examined for inhibition of primed elongation activity. PE44-NoV RNA templates (32 nucleotides) were extended (44 nucleotides) by the RdRp in the absence of any test compounds (0.5% DMSO [vol/vol] negative control) or with test compounds at a fixed concentration of 100 µM. Lane 1 of each gel: Low range ssRNA ladder (NEB). Lane 2: No RdRp with DMSO (0.5% [vol/vol]), used as a negative control. Lane 3: Norovirus RdRp with DMSO (0.5% [vol/vol]), used as a positive control. Lane 4nad 5: PPNDS and compound 1 were used as positive controls (10 0µM) to demonstrate complete inhibition.


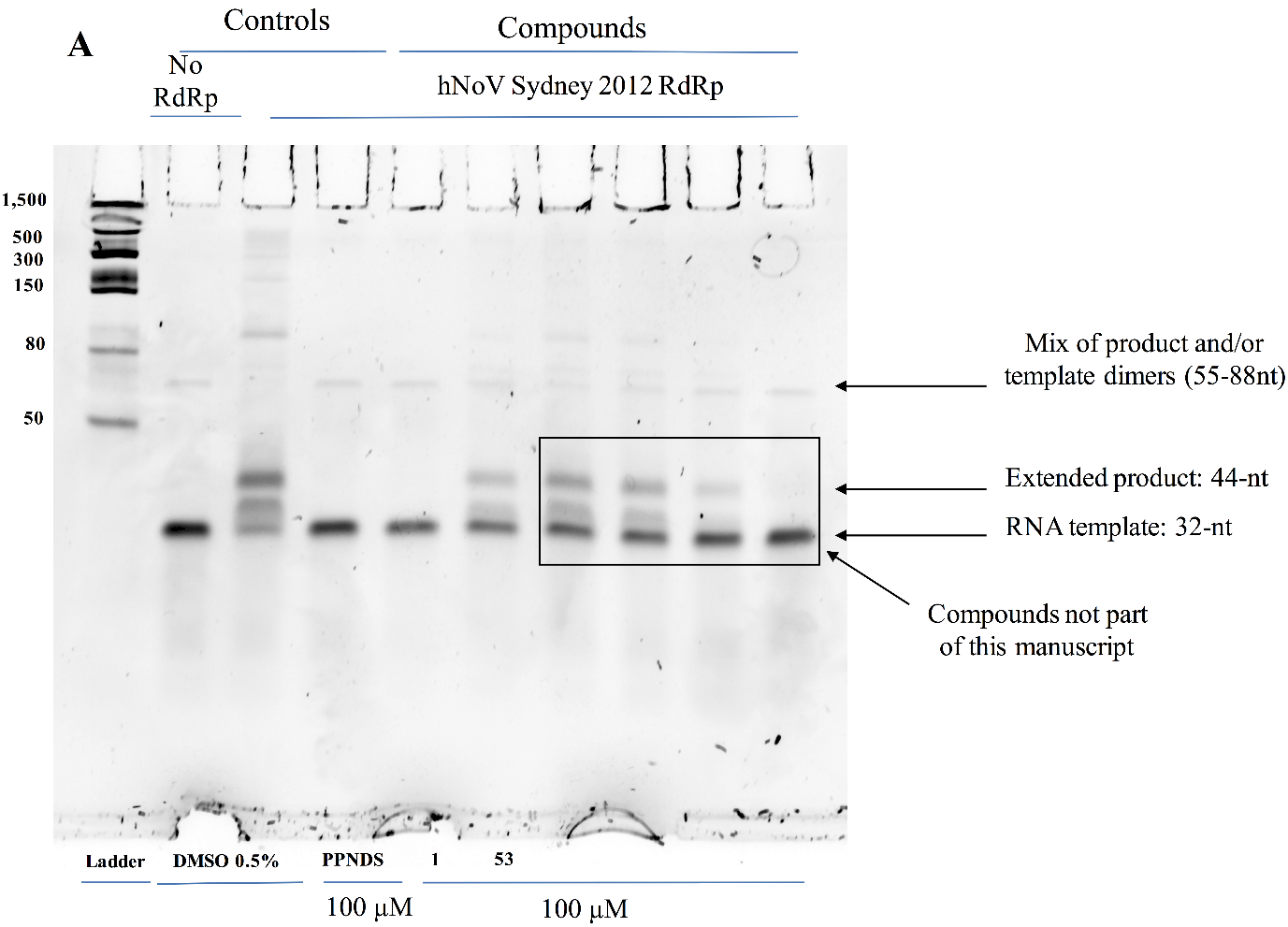


**Supplementary Figure S4. Full length gel from Figure 8.** A counter-screen gel-shift assay was used to confirm norovirus RdRp inhibitory activity of 53. The compound were examined for inhibition of primed elongation activity. PE44-NoV RNA templates (32 nucleotides) were extended (44 nucleotides) by the RdRp in the absence of any test compounds (0.5% DMSO [vol/vol] negative control) or with test compounds at a fixed concentration of 100 µM. Lane 1 of each gel: Low range ssRNA ladder (NEB). Lane 2: No RdRp with DMSO (0.5% [vol/vol]), used as a negative control. Lane 3: Norovirus RdRp with DMSO (0.5% [vol/vol]), used as a positive control. Lane 4nad 5: PPNDS and compound 1 were used as positive controls (10 0µM) to demonstrate complete inhibition.

| **Compound** | **EC_50_ values [μM]^A^** | **CC_50_ values [μM]^A^** |
| --- | --- | --- |
| **2** | >100 | >100 |
| **3** | >100 | >100 |
| **4** | >50 | 60±21 |
| **5** | >100 | >100 |
| **6** | 89±2 | >100 |
| **7** | >12.5 | 20±21 |
| **34** | >100 (crystals) | >100 |
| **35** | >100 | >100 |
| **Positive control?** |  |  |

^A^ The mean values ± standard deviations are shown from at least two independent experiments.

**Supplementary Table S1.** EC_50_ and CC_50_ values of **2-7, 34-35** in the MNV CPE reduction assay.

**S.1 General method for the preparation of ethyl esters 10-11** ^S1^

To a solution of the appropriate hydrazineylbenzoic acid (1 mmol) in EtOH (1.5 ml/mmol) was added ethanolic HCl 10% (3.5 ml/mmol) and heated 80˚C for 3 days. The reaction mixture was allowed to cool to room temperature and the solid obtained was filtered off under vacuum. The solid was dried to afford the desired ethyl hydrazineylbenzoate.

S.1.1 Ethyl 4-hydrazineylbenzoate (**10**)

Obtained in 90% yield as a pale yellow solid. ^1^H-NMR (DMSO-d_6_), δ: 10.13 (bs, 2H), 8.81 (bs, 1H), 7.87 (d, J= 8.8 Hz, 2H), 6.98 (d, J= 8.8 Hz, 2H), 4.26 (q, J= 7.1 Hz, 2H), 1.30 (t, J= 7.1 Hz, 3H). ^13^C-NMR (DMSO-d_6_), δ: 165.8, 150.2, 130.9, 122.3, 133.5, 60.7, 14.7.

S.1.2 Ethyl 3-hydrazineylbenzoate (**11**)

Obtained in 79% yield as a white solid. ^1^H-NMR (DMSO-d_6_), δ: 10.28 (bs, 2H), 8.53 (bs, 1H), 7.58-7.57 (m, 1H), 7.54-7.52 (m, 1H), 7.45-7.42 (m, 1H), 7.24-7.22 (m, 1H), 4.32 (q, J= 7.0 Hz, 2H), 1.33 (t, J= 7.0 Hz, 3H). ^13^C-NMR (DMSO-d_6_), δ: 160.0, 146.4, 131.1, 129.8, 122.4, 119.5, 114.8, 61.3, 14.6.

**S.2 Synthesis of ethyl 2-hydrazineylbenzoate 37**^S2^

Ethyl 2-aminobenzoate **36** (6.0 mmol) was dissolved in concentrated HCl (10 ml) and cooled to 0 °C. A solution of NaNO_2_ (6.4 mmol) in H_2_O (2.5 ml) was added over 15 minutes. The solution was stirred for further 2 minutes at 0 °C, after which a solution of SnCl_2_ (13.31 mmol) in concentrated HCl (3 ml) was added drop-wise. A precipitate was formed. The reaction was stirred for 3 hours at 0 °C, then the solid wad filtered, washed with ice-cold concentrated HCl and dried under *vacuum*. Obtained in 99% yield as a white solid. ^1^H-NMR (DMSO-d_6_), δ: 10.53 (bs, 2H), 8.91 (bs, 1H), 7.91 (dd, J_1_= 7.9 Hz, J_2_= 1.54 Hz, 1H), 7.63-7.59 (m, 1H), 7.18-7.16 (m, 1H), 7.04-7.01 (m, 1H), 4.32 (q J= 7.0 Hz, 2H), 1.33 (t, J= 7.0 Hz, 3H). ^13^C-NMR (DMSO-d_6_), δ: 167.0, 147.3, 135.0, 131.4, 120.7, 114.3, 113.6, 61.4, 14.5.

**S.3 General method for the preparation of ethyl 3-oxo-3-(2-arylhydrazineyl)propanoates 14-16, 38** ^S3^

The appropriate hydrazine **10-12**, **37** (1.5 mmol) was dissolved in anhydrous THF (1.1 ml/mmol) under N_2_ atmosphere and NEt_3_ (1 mmol) was added. The mixture was cooled to -10 °C and a solution of ethyl 3-chloro-3-oxopropanoate **13** (1 mmol) in anhydrous THF (4.2 ml/mmol) was added drop-wise. The mixture was allowed to worm to room temperature and stirred for 3 hours. The reaction was concentrated under vacuum and the residue obtained was extracted between EtOAc (3x20 ml) and H_2_O (10 ml). The organic layer was dried over MgSO_4_ and concentrated under vacuum. The crude product was purified by flash column chromatography to give the title compound.

S.3.1 Ethyl 4-(2-(3-ethoxy-3-oxopropanoyl)hydrazineyl)benzoate (**14**)

Purified by automated flash column chromatography (Biotage Isolera One, SNAP KP Sil 50g) eluting with *n*-hexane:EtOAc 100:0:0 v/v increasing to 0:100 v/v in 10 CV. Obtained in 75% yield as a yellow solid. ^1^H-NMR (DMSO-d_6_), δ: 10.02 (d, J= 1.5 Hz, 1H), 8.56 (d, J= 1.5 Hz, 1H), 7.75 (d, J= 8.8 Hz, 2H), 6.76 (d, J= 8.8 Hz, 2H), 4.23 (q, J= 7.1 Hz, 2H), 4.13 (q, J= 7.1 Hz, 2H), 3.35 (s, 2H), 1.28 (t, J= 7.1 Hz, 3H), 1.22 (t, J= 7.1 Hz, 3H). ^13^C-NMR (DMSO-d_6_), δ: 167.9, 166.1, 165.6, 153.4, 131.0, 119.6, 111.3, 61.2, 60.3, 41.2, 14.8, 14.5.

S.3.2 Ethyl 3-(2-(3-ethoxy-3-oxopropanoyl)hydrazineyl)benzoate (**15**)

Purified by automated flash column chromatography (Biotage Isolera One, SNAP KP Sil 25g) eluting with *n*-hexane:EtOAc 100:0:0 v/v increasing to 0:100 v/v in 10 CV. Obtained in 77% yield as a yellow solid. ^1^H-NMR (DMSO-d_6_), δ: 10.03 (d, J= 4.1 Hz, 1H), 8.68 (d, J= 4.0 Hz, 1H), 7.52-7.50 (m, 1H), 7.46-7.45 (m, 1H), 7.24-7.20 (m, 1H), 6.98-6.95 (m, 1H), 6.16 (d, J= 4.1 Hz, 1H), 4.28 (q, J= 7.1 Hz, 2H), 4.19 (q, J= 7.1 Hz, 2H), 3.39 (s, 2H), 1.30 (t, J= 7.1 Hz, 3H), 1.25 (t, J= 7.1 Hz). ^13^C-NMR (DMSO-d_6_), δ: 168.7, 165.5, 165.3, 147.9, 131.5, 129.1, 122.5, 118.0, 114.4, 62.0, 61.0, 40.3, 14.3, 14.0.

S.3.3 Ethyl 3-oxo-3-(2-phenylhydrazineyl)propanoate (**16**)

Purified by automated flash column chromatography (Biotage Isolera One, SNAP KP Sil 100g) eluting with *n*-hexane:EtOAc 100:0:0 v/v increasing to 0:100 v/v in 15 CV. Obtained in 99% yield as a yellow solid. ^1^H-NMR (DMSO-d_6_), δ: 9.83 (d, J= 2.4 Hz, 1H), 7.82 (d, J= 2.4 Hz, 1H), 7.15-7.11 (m, 2H), 6.75-6.72 (m, 2H), 6.72-6.68 (m, 1H), 4.12 (q, J= 6.8 Hz, 2H), 3.32 (s, 2H), 1.22 (t, J= 6.8 Hz, 3H). ^13^C-NMR (DMSO-d_6_), δ: 168.0, 165.7, 149.2, 129.1, 119.1, 112.6, 61.1, 41.3, 14.4.

S.3.4 Ethyl 2-(2-(3-ethoxy-3-oxopropanoyl)hydrazineyl)benzoate (**38**)

Purified by automated flash column chromatography (Biotage Isolera One, SNAP KP Sil 50g) eluting with *n*-hexane:EtOAc 100:0:0 v/v increasing to 0:100 v/v in 15 CV. Obtained in 89% yield as a yellow solid. ^1^H-NMR (DMSO-d_6_), δ: 10.21 (d, J= 2.1 Hz, 1H), 8.90 (d, J= 2.1 Hz, 1H), 7.87-7.84 (m, 1H), 7.47-7.44 (m, 1H), 7.00 (dd, J_1_= 8.4 Hz, J_2_= 1.0 Hz, 1H), 6.83-6.80 (m, 1H), 4.31 (m, 1H), 4.13 (q, J= 7.1 Hz, 2H), 3.36 (s, 2H), 1.33 (t, J= 7.1 Hz, 2H), 1.22 (t, J= 7.1 Hz, 3H). ^13^C-NMR (DMSO-d_6_), δ: 167.9, 167.5, 164.4, 151.3, 111.3, 134.9, 131.2, 118.2, 112.9, 61.2, 61.0, 41.2, 14.6, 14.5.

**S.4 General method for the preparation of 1-phenylpyrazolidine-3,5-diones 17-19** ^S-3^

The appropriate ethyl 3-oxo-3-(2-arylhydrazineyl)propanoate **14-16** (1 mmol) was dissolved in EtOH (2 ml/mmol) and an ethanolic 1M NaOH solution (2.2 ml/mmol) was added. The reaction mixture was stirred at room temperature for 30 minutes. The reaction mixture was then acidified by addition of 1M HCl solution. The precipitate formed was collected by filtration, washed with H_2_O and dried in vacuo to give the desired product. In some cases, the product did not precipitate upon acidification. The solvent was therefore removed under *vacuum* and extracted with EtOAc (3x10 ml). The organic layer was dried over MgSO_4_, evaporated under *vacuum* and purified by flash column chromatography to afford the desired product.

S.4.1 Ethyl 4-(3,5-dioxopyrazolidin-1-yl)benzoate (**17**)

Precipitated clean from the reaction mixture. Obtained in 55% yield as an orange solid. ^1^H-NMR (DMSO-d_6_), δ:

11.49 (bs, 1H), 8.00 (d, J= 8.7 Hz, 2H), 7.90-7.77 (m, 2H), 4.30 (q, J= 7.1 Hz, 2H), 3.67 (s, 2H), 1.32 (t, J= 7.1 Hz, 3H). ^13^C-NMR (DMSO-d_6_), δ: 176.6, 176.1, 176.0, 165.9, 165.6, 130.7, 118.3, 61.1, 38.3, 14.6.

S.4.2 Ethyl 3-(3,5-dioxopyrazolidin-1-yl)benzoate (**18**)

Purified by automated flash column chromatography (Biotage Isolera One, SNAP KP Sil 50g) eluting with EtOAc:MeOH 100:0 v/v increasing to 80:20 v/v in 15 CV. Obtained in 43% yield as a yellow solid. ^1^H-NMR (CDCl_3_), δ: 8.06-8.02 (m, 1H), 7.85-7.82 (m, 1H), 7.44-7.41 (m, 1H), 4.30 (q, J= 7.1 Hz, 2H), 3.42 (s, 2H), 1.32 (t, J= 7.1 Hz, 3H). ^13^C-NMR (CDCl_3_), δ: 164.0, 161.0, 159.2, 131.0, 126.9, 124.7, 122.2, 118.7, 114.3, 56.7, 32.9, 9.5.

S.4.3 1-Phenylpyrazolidine-3,5-dione (**19**)

Purified by automated flash column chromatography (Biotage Isolera One, SNAP KP Sil 50g) eluting with DCM:MeOH 100:0 v/v increasing to 90:10 v/v in 10 CV. Obtained in 33% yield as a yellow solid. ^1^H-NMR (DMSO-d_6_), δ: 11.48 (bs, 1H), 7.67-7.60 (m, 2H), 7.43-7.39 (m, 2H), 7.29-7.14 (m, 1H), 3.59 (s, 2H). ^13^C-NMR (DMSO-d_6_), δ: 176.7, 144.2, 128.9, 125.0, 118.7, 38.3.

**S.5 General method for the preparation of 4-bromo-*N*-arylsulfonamides 25-28** ^S4^

4-Bromobenzenesulfonyl chloride **24** (1.2 mmol) dissolved in 1.5 ml/mmol of pyridine was added drop-wise at 0 °C to a stirring solution of the differently substituted aniline **20-23** (1 mmol) in anhydrous pyridine (0.5 ml/mmol). The reaction was stirred overnight at room temperature. EtOAc (15 ml) was added to the reaction mixture, which was then washed with 2M aqueous HCl (10 ml) and brine (10 ml). The organic layer was dried over Na_2_SO_4_ and evaporated under vacuum. The crude products were purified by precipitation or flash column chromatography. S.5.1 4-Bromo-*N*-phenylbenzenesulfonamide (**25**)

Purified by re-crystallisation from EtOAc/*n*-hexane. Obtained in 81% yield as a yellow solid. ^1^H-NMR (DMSO-d_6_), δ: 10.35 (bs, 1H), 7.77 (d, J= 8.7 Hz, 2H), 7.66 (d, J= 8.7 Hz, 2H), 7.26-7.22 (m, 2H), 7.09-7.03 (m, 3H). ^13^C-NMR (DMSO-d_6_), δ: 139.2, 137.8, 127.2, 132.8, 129.1, 126.7, 124.8, 120.8.

S.5.2 4-Bromo-*N*-(pyrimidin-2-yl)benzenesulfonamide (**26**)

Purified by re-crystallisation from DCM/*n*-hexane. Obtained in 87% yield as a yellow solid. ^1^H-NMR (DMSO-d_6_), δ: 12.03 (bs, 1H), 8.52-8.50 (m, 2H), 7.90 (d, J= 8.7 Hz, 2H), 7.80 (d, J= 8.7 Hz, 2H), 7.08-7.05 (m, 1H).

^13^C-NMR (DMSO-d_6_), δ: 157.0, 140.2, 132.4, 131.1, 130.0, 128.2, 127.2, 116.2.

S.5.3 4-Bromo-*N*-(oxazol-2-yl)benzenesulfonamide (**27**)

Purified by automated flash column chromatography (Biotage Isolera One, SNAP KP Sil 50g) eluting with *n*-hexane:EtOAc 100:0 v/v increasing to 0:100 v/v in 10 CV. Obtained in 68% yield as an orange solid. ^1^H-NMR (CDCl_3_), δ: 10.44 (bs, 1H), 7.75 (d, J= 8.7 Hz, 2H), 7.54 (d, J= 8.7 Hz, 2H), 7.39 (d, J= 6.8 Hz, 1H), 6.88 (d, J= 6.8 Hz, 1H). ^13^C-NMR (CDCl_3_), δ: 156.6, 142.8, 132.4, 128.5, 126.0, 116.3, 114.7.

S.5.4 *N*-(1H-Benzo[d]imidazol-2-yl)-4-bromobenzenesulfonamide (**28**)

Purified by trituration from DCM. Obtained in 85% yield as a white solid. ^1^H-NMR (DMSO-d_6_), δ: 7.96 (d, J= 8.8 Hz, 2H), 7.88 (d, J= 8.8 Hz, 2H), 7.66 (m, 1H), 7.20-7.19 (m, 2H), 7.18-7.18 (m, 1H), 7.15-7.14 (m, 1H), 7.05-7.04 (m, 1H). ^13^C-NMR (DMSO-d_6_), δ: 152.4, 143.2, 136.2, 133.6, 130.4, 130.1, 129.1, 125.3, 121.1, 116.5, 112.6.

**S.6 Synthesis of 4-(5-formylfuran-2-yl)-*N*-phenylbenzenesulfonamide 30**^S5^

A solution of (5-formylfuran-2-yl)boronic acid **29** (3.2 mmol), 4-bromo-*N*-phenylbenzenesulfonamide **25** (3.2 mmol), K_3_PO_4_ (3.2 mmol) and Pd(dppf)Cl_2_ (0.096 mmol) in a mixed solvent of H_2_O (1.5 ml) and DMF (6 ml) was heated under microwave irradiation at 130 °C for 75 min. The mixture was then diluted with DCM (15 ml) and washed with saturated aqueous NaHCO_3_ solution (10 ml). The organic layer was dried over MgSO_4_ and concentrated under *vacuum*. The crude product was purified by automated flash column chromatography (Biotage Isolera One, SNAP KP Sil 10g) eluting with DCM:MeOH 100:0 v/v increasing to 95:5 v/v in 10 CV. Obtained in 45% yield as an off-white solid. ^1^H-NMR (DMSO-d_6_), δ: 10.34 (bs, 1H), 9.65 (s, 1H), 8.02 (d, J= 8.7 Hz, 2H), 7.85 (d, J= 8.7 Hz, 2H), 7.67 (d, J= 3.7 Hz, 1H), 7.43 (d, J= 3.7 Hz, 1H), 7.26-7.22 (m, 2H), 7.11-7.09 (m, 2H), 7.06-7.02 (m, 1H). ^13^C-NMR (DMSO-d_6_), δ: 179.0, 156.4, 152.7, 140.3, 138.0, 132.7, 129.7, 128.0, 126.0, 124.7, 120.9, 111.5.

**S.7 General method for the preparation of furaldehydes 31-33**

Pd(OAc)_2_ (0.005 mmol) and PPh_3_ (0.01 mmol) were added to a suspension of the differently substituted benzenesulfonamide **26-28** (1 mmol), (5-formylfuran-2-yl)boronic acid **29** (1 mmol) and Na_2_CO_3_ (3 eq) in a mixture of DME (1ml/mmol) and H_2_O (0.5 ml/mmol). The reaction was stirred under microwave irradiation at 85 °C for 10 minutes. The solvent was evaporated, and the residue was taken up in water (10 ml) and extracted with DCM (20 ml). The aqueous layer was acidified with 2N aqueous HCl and extracted with DCM (3x 20 ml). The combined organic layers were dried over MgSO_4_ and evaporated under vacuum. The crude products were purified by flash column chromatography to give the desired compounds.

S.7.1 4-(5-formylfuran-2-yl)-N-(pyrimidin-2-yl)benzenesulfonamide (**31**)

Purified by automated flash column chromatography (Biotage Isolera One, SNAP KP Sil 25g) eluting with EtOAc:MeOH 100:0 v/v increasing to 80:20 v/v in 15 CV. Obtained in 51% yield as an off-white solid. ^1^H-NMR (DMSO-d_6_), δ: 10.34 (bs, 1H), 9.65 (s, 1H), 8.52-8.49 (m, 2H), 8.09 (d, 8.7 Hz, 2H), 8.05 (d, J= 8.7 Hz, 2H), 7.68 (d, J= 3.7 Hz, 1H), 7.45 (d, J= 3.7 Hz, 1H), 7.07-7.03 (m, 1H).

S.7.2 4-(5-formylfuran-2-yl)-N-(oxazol-2-yl)benzenesulfonamide (**32**)

Purified by automated flash column chromatography (Biotage Isolera One, SNAP KP Sil 10g) eluting with EtOAc:MeOH 100:0 v/v increasing to 90:10 v/v in 10 CV. Obtained in 42% yield as a pale yellow solid. ^1^H-NMR (DMSO-d_6_), δ: 12.22 (bs, 1H), 9.65 (s, 1H), 8.02 (d, J= 8.6 Hz, 1H), 7.96 (d, J= 8.6 Hz, 1H), 7.69 (d, J= 3.7 Hz, 1H), 7.62 (d, J= 1.5 Hz, 1H), 7.44 (d, J= 3.7 Hz, 1H), 7.29 (d, J= 1.5 Hz, 1H). ^13^C-NMR (DMSO-d_6_), δ: 178.7, 176.6, 157.0, 152.6, 131.9, 129.2, 129.1, 128.5, 127.4, 125.7, 118.2, 111.8, 111.0.

S.7.3 *N*-(1H-Benzo[d]imidazol-2-yl)-4-(5-formylfuran-2-yl)benzenesulfonamide (**33**)

Purified by automated flash column chromatography (Biotage Isolera One, SNAP KP Sil 25g) eluting with EtOAc:MeOH 100:0 v/v increasing to 80:20 v/v in 10 CV. Obtained in 34% yield as a yellow solid. ^1^H-NMR (DMSO-d_6_), δ: 9.65 (s, 1H), 8.15 (d, J= 8.8 Hz, 2H), 8.09 (d, J= 8.8 Hz, 2H), 7.70-7.68 (m, 1H), 7.67 (d, J= 3.7 Hz, 1H), 7.49 (d, J= 3.7 Hz, 1H), 7.20-7.19 (m, 2H), 7.15-7.13 (m, 2H), 7.05-7.02 (m, 1H). ^13^C-NMR (DMSO-d_6_), δ: 182.8, 176.6, 155.8, 152.4, 143.2, 136.8, 134.9, 131.9, 130.4, 129.2, 128.2, 126.5, 125.3, 121.1, 116.5, 112.6.

**S.8 Synthesis of *N*-(4-(*N*-phenylsulfamoyl)phenyl)acetamide 42**^S6^

*N*-Acetylsulfanyl chloride **41** (2.1 mmol) was added in portion to a stirred solution of aniline **40** (2.6 mmol) in pyridine (4 mL), while cooling the reaction mixture at 0 °C. The reaction was stirred at 0 °C for 4 h, then water (15 mL) was added, and the mixture was extracted with EtOAc (25 mL). The organic layer was washed with 2N aqueous HCl solution (20 mL) and brine (15 mL), dried over Na_2_SO_4_, concentrated under *vacuum*, and used for the next step without further purification. Obtained in 91% yield as an off-white solid. ^1^H-NMR (DMSO-d_6_), δ: 10.28 (bs, 1H), 10.14 (bs, 1H), 7.68-7.68 (d, 2H), 7.23-7.20 (m, 2H), 7.07 (d, J= 8.3 Hz, 2H), 7.03-6.97 (m, 3H), 2.06 (s, 3H).

**S.9 Synthesis of 4-amino-*N-*phenylbenzenesulfonamide 43**^S6^

*N*-(4-(*N*-phenylsulfamoyl)phenyl)acetamide **42** (1.6 mmol) was suspended in a mixture of 3 mL of 5M aqueous NaOH and 2 mL of MeOH. The mixture was stirred at 70 °C for 3 h, then extracted with EtOAc (30 mL). The organic layer was washed with saturated aqueous NaHCO_3_ solution (20 mL) and brine (15 mL), dried over Na_2_SO_4_, concentrated under *vacuum*, and used for the next step without further purification. Obtained in 89% yield as a light brown solid. ^1^H-NMR (DMSO-d_6_), δ: 9.83 (bs, 1H), 7.38 (d, J= 8.7 Hz, 2H), 7.21-7.18 (m, 2H), 7.07-7.04 (m, 2H), 7.01-6.95 (m, 1H), 6.52 (d, J= 8.7 Hz, 2H), 5.94 (bs, 2H).

**S.10 Synthesis of methyl 2-([1,1'-biphenyl]-3-yl)acetate 46**^S7^

Phenylboronic acid **45** (1.4 mmol) and methyl 2-(3-bromophenyl)acetate **44** (1.3 mmol) were dissolved in toluene (9 mL). A solution of Na_2_CO_3_ (2.9 mmol) in water (3mL) was then added, along with EtOH (3 mL), followed by tetrakis palladium (0.05 mmol). The mixture was stirred overnight at 100 °C, then cooled to room temperature and partitioned between EtOAc (20 mL) and 1M aqueous NaOH solution (15 mL). The organic layer was washed with brine, dried over Na_2_SO_4_, and concentrated under *vacuum*. The crude residue was purified by automated flash column chromatography (Biotage Isolera One, SNAP KP Sil 10g) eluting with *n*-hexane:EtOAc 100:0 v/v increasing to 50:50 v/v in 10 CV. Obtained in 75% yield as an off-white solid. ^1^H-NMR (CDCl_3_), δ: 7.51-7.49 (m, 2H), 7.42-7.41 (m, 2H), 7.36-7.30 (m, 3H), 7.27-7.25 (m, 1H), 7.19-7.16 (m, 1H), 3.62 (s, 3H), 3.61 (s, 2H).

**S.11 Synthesis of 2-([1,1'-biphenyl]-3-yl)acetic acid 47**^S7^

Methyl 2-([1,1'-biphenyl]-3-yl)acetate **46** (0.59 mmol) was dissolved in MeOH (4 mL) and added of a solution of LiOH (1.1 mmol) in water (0.5 mL). The mixture was stirred at room temperature for 2 hours, then concentrated under *vacuum* to remove the MeOH. The aqueous residue was acidified with 2N HCl and extracted with DCM (3x20 mL). The combined organic layers were dried over Na_2_SO_4_ and concentrated under *vacuum* to afford the title compound in 793% yield as an off-white solid. ^1^H-NMR (DMSO-d_6_), δ: 10.70 (bs, 1H), 7.51-7.49 (m, 2H), 7.44-7.43 (m, 2H), 7.37-7.31 (m, 3H), 7.28-7.25 (m, 1H), 7.20-7.18 (m, 2H), 3.64 (s, 2H).

**S.12 General method for the preparation of amides 62a-65a**

1-(4-Nitrophenyl)piperazine **61** (2.4 mmol), the appropriately substituted carboxylic acid (2.6 mmol) and TBTU (2.8 mmol) were dissolved in dry DMF (7 mL) under N_2_ atmosphere. Then, DiPEA (6.0 mmol) was added to the mixture and the reaction was stirred at room temperature for 5h. The solution was then partitioned between EtOAc (100 mL) and saturated aqueous NH_4_Cl (70 mL). The organic phase was re-extracted once with NH_4_Cl (70 mL), dried over Na_2_SO_4_ and concentrated under vacuum. The crude residue was purified by flash column chromatography or trituration to afford the desired compounds.

S.12.1 (4-(4-Nitrophenyl)piperazin-1-yl)(thiophen-2-yl)methanone (**62a**)^S-8^

Purified by trituration from EtOAc/*n*-hexane. Obtained in 94% yield as a yellow solid. ^1^H-NMR (DMSO-d_6_), δ: 8.09 (d, J=9.5 Hz, 2H), 7.80 (dd, J_1_=6.0 Hz, J_2_=1.1 Hz, 1H), 7.50 (dd, J_1_=3.7 Hz, J_2_=1,1 Hz, 1H), 7,17 (dd, J_1_=6.0 Hz, J_2_=3.7 Hz, 1H), 7.00 (d, J=9.5 Hz, 2H), 3.83- 3.82 (m, 4H), 3.63-3.61 (m, 4H).

S.12.2 Furan-2-yl(4-(4-nitrophenyl)piperazin-1-yl)methanone (**63a**)^S-9^

Purified by trituration from EtOAc/*n*-hexane. Obtained in 81% yield as a yellow solid. ^1^H-NMR (DMSO-d_6_), δ: 8.09 (d, J= 9.5 Hz), 7.88 (dd, J_1_= 1.8 Hz, J_2_= 0.8 Hz, 1H), 7.07 (dd, J_1_= 4.1 Hz, J_2_=0.8 Hz, 1H), 7.02 (d, J= 9.5 Hz, 2H), 6.66 (dd, J_1_= 4.1 Hz, J_2_= 1.8 Hz, 1H), 3.85 (m, 4H), 3.61-3.59 (m, 4H).

S.12.3 (4-(4-Nitrophenyl)piperazin-1-yl)(phenyl)methanone (**64a**)^S-10^

Purified by trituration from EtOAc/*n*-hexane. Obtained in 99% yield as an orange solid. ^1^H-NMR (DMSO-d_6_), δ: 8.10-8.07 (m, 2H), 7.50-4.45 (m, 5H), 7.04-7.01 (m, 2H), 3.76-3.51 (m, 8H). ^13^C-NMR (DMSO-d_6_), δ: 169.6, 154.8, 137.5, 136.1, 130.2, 128.9, 127.5, 126.2, 113.2, 31.4, 22.5.

S.12.4 (4-(4-Nitrophenyl)piperazin-1-yl)(thiazol-2-yl)methanone (**65a**)

Purified by automated flash column chromatography (Biotage Isolera One, SNAP KP Sil 25g) eluting with DCM:MeOH 100:0 v/v increasing to 90:10 v/v in 15 CV. Obtained in 99% yield as an off-white solid. ^1^H-NMR (CDCl_3_), δ: 8.20-8.17 (m, 2H), 7.94 (d, J= 3.2 Hz, 1H), 7.61 (d, J= 3.2 Hz, 1H), 6.90-6.86 (m, 2H), 4.68-4.67 (m, 2H), 4.03-4.01(m, 2H), 3.60 (m, 4H).

**S.13 General method for the preparation of sulfonamides 66a-67a**

1-(4-Nitrophenyl)piperazine **61** (2.4 mmol) was dissolved in DCM (15 mL), Net_3_ (2.8 mmol) was added and the reaction was cooled to 0 °C. The appropriate arylsulfonyl chloride (2.6 mmol) was dissolved in 5 mL DCM and the solution was added drop-wise to the reaction mixture. The reaction was stirred at 0 °C for 1 hour, then allowed to warm to room temperature and stirred overnight. Upon completion of the reaction, saturated aqueous NaHCO_3_ (20 mL) was added and the reaction was extracted with DCM (3x 25 mL). The organic phase was evaporated at reduced pressure and the crude residue was purified by flash column chromatography or trituration to afford the desired compounds.

S.13.1 4-(4-(Thiophen-2-ylsulfonyl)piperazin-1-yl)aniline (**66a**)

Purified by trituration from EtOAc/n-hexane. Obtained in 99% yield as a yellow solid. ^1^H-NMR (DMSO-d_6_), δ: 8.08 (dd, J_1_= 5.3 Hz, J_2_= 1.3 Hz, 1H), 8.05 (dd, J_1_= 5.3 Hz, J_2_= 3.8 Hz, 2H), 7.68 (dd, J_1_= 3.8Hz, J_2_= 1.3 Hz, 1H), 7.32-7.30 (m, 2H), 7.04-7.01 (m, 2H) 3.60 (m, 4H), 3.06 (m, 4H). ^13^C-NMR (DMSO-d_6_), δ: 154.6, 138.1, 135.0, 134.7, 133.8, 128.9, 126.1, 113.7, 46.2, 45.8.

S.13.2 1-(4-Nitrophenyl)-4-(phenylsulfonyl)piperazine (**67a**)

Purified by automated flash column chromatography (Biotage Isolera One, SNAP KP Sil 25g) eluting with DCM:MeOH 100:0 v/v increasing to 90:10 v/v in 15 CV. Obtained in 89% yield as a yellow solid. ^1^H-NMR (DMSO-d_6_), δ: 8.05-8.02 (m, 2H), 7.79-7.76 (m, 3H), 7.69-7.65 (m, 2H), 7.01-6.98 (m, 2H), 3.56 (m, 4H), 3.02 (m, 4H).^13^C-NMR (DMSO-d_6_), δ: 154.6, 137.9, 135.1, 133.9, 130.0, 128.1, 126.1, 113.7, 46.3, 45.8.

**S.14 General method for the preparation of aromatic amines 62b-67b**

The corresponding nitro compound **62a-67a** (1 mmol) was dissolved in EtOH (15 mL/mmol) and added of 10% wet Pd/C (0.1 g/mmol). The suspension was stirred under a H_2_ atmosphere and stirred at room temperature for 24 hours. The black suspension was diluted with 1:1 DCM/MeOH solution and then was filtered through celite under *vacuum*. The filtrate was evaporated at reduced pressure to afford the title compound which was used for the next step without further purification unless otherwise stated.

S.14.1 (4-(4-Aminophenyl)piperazin-1-yl)(thiophen-2-yl)methanone (**62b**)^S-8^

Obtained in 92% yield as a light brown solid. ^1^H-NMR (DMSO-d_6_), δ: 7.82 (dd, J_1_= 7.2 Hz, J_2_= 1.1 Hz, 1H), 7.49 (dd, J_1_= 4.2 Hz, J_2_= 1.1 Hz, 1H), 7,19 (dd, J_1_= 7.2 Hz, J_2_= 4.2 Hz, 1H) 6.78 (d J= 8.9 Hz, 2H), 6.56 (d, J=8.9 Hz, 2H), 4.68 (bs, 2H), 3.82-3.80 (m, 4H), 3.01-2.99 (m, 4H). ^13^C-NMR (DMSO-d_6_), δ: 162.7, 143.3, 142.4, 137.6, 129.9, 129.6, 127.6, 119.2, 115.2, 51.4.

S.14.2 (4-(4-Aminophenyl)piperazin-1-yl)(furan-2-yl)methanone (**63b**)^S-9^

Obtained in 99% yield as a pink solid. ^1^H-NMR (DMSO-d_6_), δ: 7.91 (dd, J_1_= 1.8 Hz, J_2_= 0.8 Hz, 1H), 7.07 (dd, J_1_= 3.9 Hz, J_2_= 0.8 Hz, 1H), 6.78 (d, J= 8.8 Hz, 2H), 6.69 (dd, J_1_= 3.9 Hz, J2=1.8 Hz, 1H), 6.58-6.55 (m, 2H), 4.68 (bs, 2H), 3.83 (m, 4H), 3.01-2.99 (m, 4H). ^13^C-NMR (DMSO-d_6_), δ: 158.7, 147.5, 145.2, 143.3, 142.4, 119.6, 116.1, 115.2, 111.8, 56.5.

S.14.3 (4-(4-Aminophenyl)piperazin-1-yl)(furan-2-yl)methanone (**64b**)^S-11^

Obtained in 78% yield as a light brown solid. ^1^H-NMR (DMSO-d_6_), δ: 7.48-7.41 (m, 5H), 6.73-6.70 (m, 2H), 6.52-6.49 (m, 2H), 3.74 (bs, 2H), 3.43-3.38 (m, 8H). ^13^C-NMR (DMSO-d_6_), δ: 169.4, 143.2, 142.4, 136.4, 129.9, 128.9, 127.4, 119.2, 115.1, 51.2.

S.14.4 (4-(4-Aminophenyl)piperazin-1-yl)(furan-2-yl)methanone (**65b**)

Obtained in 80% yield as a white solid. ^1^H-NMR (CDCl_3_), δ: 7.92 (d, J=3.2 Hz, 2H),7.57 (d, J=3.2 Hz, 2H), 6.87-6.84 (m, 2H), 6.71-6.67 (m, 2H), 4.60-4.59 (m, 2H), 3.99-3.97 (m, 2H), 3.16-3.14 (m, 4H). ^13^C-NMR (CDCl_3_), δ: 165.2, 159.2, 144.0, 143.1, 140.8, 124.1, 119.3, 116.2, 51.8, 51.3, 46.5, 43.6.

S.14.5 4-(4-(Thiophen-2-ylsulfonyl)piperazin-1-yl)aniline (**66b**)

Obtained in 79% yield as a light brown solid. ^1^H-NMR (DMSO-d_6_), δ: 8.12 (dd, J_1_= 5.3 Hz, J_2_= 1.3 Hz, 1H), 7.72 (dd, J_1_= 3.7 Hz, J_2_= 1.3 Hz, 1H), 7.36 (dd, J_1_= 5.3 Hz, J_2_= 3.7 Hz, 1H), 6.73- 6.72 (m, 2H), 6.55-6.53 (m, 2H), 4.65 (bs, 2H), 3.11-3.10 (m, 4H), 3.05-3.04 (m, 4H). ^13^C-NMR (DMSO-d_6_), δ: 143.5, 141.9, 134.9, 133.8, 128.9, 126.1, 119.3, 115.1, 50.4, 46.6.

S.14.6 4-(4-(Phenylsulfonyl)piperazin-1-yl)aniline (**67b**)

Obtained in 88% yield as a light yellow solid. ^1^H-NMR (DMSO-d_6_), δ: 7.78-7.74 (m, 3H), 7.70-7.67 (m, 2H), 6.66-6.63 (m, 2H), 6.48-6.45 (m, 2H), 4.62 (bs, 2H), 3.00- 2.94 (m, 8H). ^13^C-NMR (DMSO-d_6_), δ: 143.5, 141.9, 135.1, 133.8, 129.9, 128.1, 119.3, 115.1, 50.5, 46.6.

**References**

**S1** Verma, A. K; Malhotra, S.; Marimganti, S.; Ray, A.; Gupta, S.; Srivastava, P.; Dastidar, S. G. Preparation of pyrazolylalkylamide derivatives for use as 5-lipoxygenase inhibitors. **2011**, WO 2011161615.

**S2** Su, G.; Gao, Y.; Sun, G.; Ren, Y. Preparation of pyrazolobenzoxazinone derivatives useful for treating cardiovascular and cerebrovascular diseases. **2008**, WO 2010006485.

**S3** Houille, O.; Fretz, H.; Hilpert, K.; Riederer, M.; Giller, T.; Valdenaire, O. Preparation of pyrazolidinedione derivatives and their use as platelet aggregation inhibitors. **2005**, WO 2005002574.

**S4** Natarajan, A.; Guo, Y.; Harbinski, F.; Fan, Y.; Chen, H.; Luus, L.; Diercks, J.; Aktas, H.; Chorev, M.; Halperin, J. A. Novel arylsulfoanilide-oxindole hybrid as an anticancer agent that inhibits translation initiation. *J. Med. Chem.* **2004**, 47, 4979-4982.

**S5** Liu, J.; Wang, K.; Zhang, X.; Li, C.; You, X. Triazine dyes as photosensitizers for dye-sensitized solar cells. *Tetrahedron.* **2013**, 69, 190-200.

**S6** Wang, L.; Li, L.; Zhou, Z.-H.; Jiang, Z.-Y.; You, Q.-D.; Xu, X.-L. Structure-based virtual screening and optimization of modulators targeting Hsp90-Cdc37 interaction. *Eur. J. Med. Chem.* **2017**, 136, 63-73.

**S7** Lessene, G. L.; Baell, J. Preparation of amino acid derivatives as α-helical mimetics. **2006**, WO 2006002474.

**S8** Al-Horani, R.A.; Mehta, A.Y.; Desai, U.R. Potent direct inhibitors of factor Xa based on the tetrahydroisoquinoline scaffold. *Eur. J. Med. Chem.* **2012**, 54, 771-783.

**S9** De Vita, D.; Moraca, F.; Zamperini, C.; Pandolfi, F.; Di Santo, R.; Matheeussen, A.; Maes, L.; Tortorella, S.; Scipione, L. In vitro screening of 2-(1H-imidazol-1-yl)-1-phenylethanol derivatives as antiprotozoal agents and docking studies on Trypanosoma cruzi CYP51. *Eur. J. Med. Chem.* **2016**, 113, 28-33.

**S10** Wodtke, R.; Steinberg, J.; Koeckerling, M.; Loeser, R.; Mamat, C. NMR-based investigations of acyl-functionalized piperazines concerning their conformational behavior in solution. *RSC Advances* **2018**, 8, 40921-40933.

**S11** Kumari, S.; Mishra, C. B.; Tiwari, M. Design, synthesis and pharmacological evaluation of N-[4-(4-(alkyl/aryl/heteroaryl)-piperazin-1-yl)-phenyl]-carbamic acid ethyl ester derivatives as novel anticonvulsant agents. *Bioorg. Med. Chem. Lett.* **2015**, 25, 1092-1099.
